# State dependence and temporal evolution of resistance in projected phase change memory

**DOI:** 10.1038/s41598-020-64826-3

**Published:** 2020-05-19

**Authors:** Benedikt Kersting, Vladimir Ovuka, Vara Prasad Jonnalagadda, Marilyne Sousa, Valeria Bragaglia, Syed Ghazi Sarwat, Manuel Le Gallo, Martin Salinga, Abu Sebastian

**Affiliations:** 1 IBM Research – Zurich, Säumerstrasse 4, 8803 Rüschlikon, Switzerland; 2 Institut für Materialphysik; Westfälische Wilhelms-Universität Münster, Wilhelm-Klemm-Straße 10, 48149 Münster, Germany

**Keywords:** Information storage, Electronic devices

## Abstract

Phase change memory (PCM) is being actively explored for in-memory computing and neuromorphic systems. The ability of a PCM device to store a continuum of resistance values can be exploited to realize arithmetic operations such as matrix-vector multiplications or to realize the synaptic efficacy in neural networks. However, the resistance variations arising from structural relaxation, 1/f noise, and changes in ambient temperature pose a key challenge. The recently proposed projected PCM concept helps to mitigate these resistance variations by decoupling the physical mechanism of resistance storage from the information-retrieval process. Even though the device concept has been proven successfully, a comprehensive understanding of the device behavior is still lacking. Here, we develop a device model that captures two key attributes, namely, resistance drift and the state dependence of resistance. The former refers to the temporal evolution of resistance, while the latter refers to the dependence of the device resistance on the phase configuration of the phase change material. The study provides significant insights into the role of interfacial resistance in these devices. The model is experimentally validated on projected PCM devices based on antimony and a metal nitride fabricated in a lateral device geometry and is also used to provide guidelines for material selection and device engineering.

## Introduction

In recent years, phase change memory (PCM) has emerged as the most mature resistive memory technology^1,2^. Besides information storage, PCM can be used for performing computational tasks. For these applications, the devices that can be programmed to arbitrary resistance/conductance states are arranged in a crossbar architecture. By exploiting Ohm’s law and Kirchhoff’s current summation rule, vector-matrix multiplications can be performed in the analog domain at potential O(1) time complexity. This computational scheme has been shown to accelerate for example linear solvers^3^, compressed sensing and image processing^4^. In the field of neuromorphic computing, training and inference of classical deep neural networks based on PCM has been demonstrated^5–7^ as well as potential applications in spiking neural networks^8–10^.

In PCM devices, a nanoscopic volume of phase change material is switched via Joule heating between two structural phases (amorphous and crystalline) of contrasting electrical properties at the nanosecond timescale. The device resistance increases monotonically with increasing amounts of material that is brought to the amorphous state. While, in theory, this should enable reliable data storage across several multi-level states, a few challenges that are inherent to the amorphous state arise. These include resistance drift^11,12^ and electrical read noise, such as the 1/f and random telegraph noise^13–15^ which induce temporal variations in the device resistance. The atomic configuration of the non-equilibrium amorphous reset state created by melt-quenching relaxes structurally towards the meta-stable super-cooled liquid state^16–18^. This structural relaxation causes a drift of resistance which can be described by the expression, $R={R}_{0}\ast {(t/{t}_{0})}^{{v}_{R}}$, where R is the instantaneous device resistance at time t, R_0_ is the resistance at time t_0_ after the creation of the reset state, and *v*_*R*_ is the drift coefficient. These inherent device non-idealities limit the multi-level storage capability of PCM.

A device that is less prone to drift and read noise can significantly improve the device performance for the afore-mentioned applications. Existing approaches to minimize these undesired traits include both materials engineering and device engineering solutions. Among the latter, a promising approach is the concept of projected PCM^19–21^. This concept decouples the device readout from the unstable electrical properties of the amorphous phase. For this purpose, an electrically conducting material, called the projection layer, is placed in parallel to the phase change material. The sheet resistance of the projection layer is chosen to lie between the sheet resistance of the crystalline and the amorphous states of the phase change material. In a projected memory device, the majority of read current bypasses the amorphized volume in the device and flows in the projection layer instead. Hence the resulting device resistance can be viewed as a projection of the length of the amorphous region onto the projection layer.

Projected PCM devices have been shown to significantly reduce resistance drift and 1/f noise, by at least an order of magnitude, thereby enabling arithmetic operations with high precision^22^. While these desired characteristics have been demonstrated through proof-of-concept memory devices, a comprehensive understanding of the device behavior is still lacking. In particular, there is a need for device models that can describe the resistance drift and the state-dependence of the resistance corresponding to the different reset states. Such models are crucial to both better understanding and building better performing projected PCM devices.

In this article, a comprehensive device model is developed to capture the behavior of the memory device for any arbitrary device state. Here device state refers to the phase configuration of the phase change material i.e. the amount of crystalline and amorphous volume in the device. We identify the interface resistance between the phase change material and projection layer as a decisive parameter. It hinders the current flow into the projection layer and thus determines the fraction of read current that bypasses the amorphous volume. Thus, it defines how effectively the projection works. Dependent on the interface resistance, key device metrics are studied. Both drift characteristics and state-dependence of the device resistance are influenced by the interface resistance and show pronounced differences with respect to an unprojected device. State-dependence refers to the change of device resistance with the size of the amorphous volume created in the device. The model is experimentally validated on nanoscale PCM devices based on pure antimony, both with and without the projection layer. The proposed modeling framework is finally extended to identify guidelines for materials selection to optimize projected PCM device characteristics.

## Device Model

**Figure 1:**
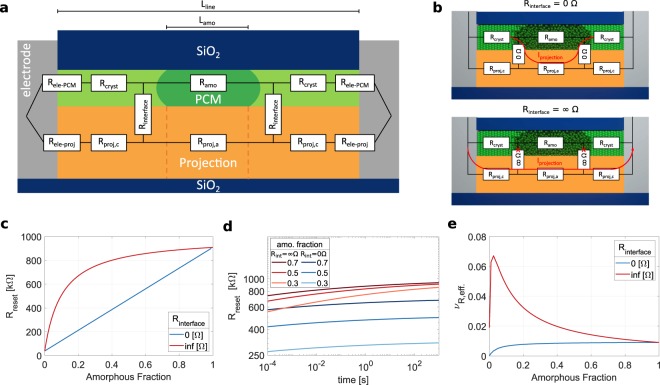
The device model and the role of interface resistance: (**a**) Sketch of a projected line-cell in cross sectional view. The equivalent circuit model of the device is depicted as a resistor network overlaying the sketch. (**b**) Top: For the scenario of a zero-interface resistance, the projection current mostly bypasses the amorphous region. Bottom: If the interface resistance is infinite the projection current bypasses the entire phase change layer (**c**) The reset resistance as a function of the amorphous fraction as predicted by the model for the two extreme cases of zero and infinite interface resistance. (**d**) The temporal evolution of resistance for different reset states as predicted by the model. (**e**) The effective drift coefficient of the device 1 s after RESET.

A device model for a projected PCM device with a symmetric, lateral device geometry (line-cell) is developed. It is assumed that during RESET the amorphous regions are created close to the center of the device (illustrated in Fig. 1a). This has been reported for lateral device structures from both, FEM simulations, as well as TEM studies^19,23,24^. Minor shifts from the center of the confined device region can occur due to thermoelectric effects, but such shifts typically remain within the confined region of the device^23,25^. An equivalent electrical circuit of this device during the low-field read operation can be described as a network of resistors, corresponding to the resistances of the crystalline and the amorphous region (R_cryst_ & R_amo_) in the device, and the projection layer resistors in parallel to them (R_proj,c_ & R_proj,a_). These resistors will be determined by the material’s sheet resistances, the device length (L_line_) and width (w) and the length of the amorphous region (L_amo_) in the device as given by,

1 $${R}_{cryst}={R}_{s,cryst}\ast ({L}_{line}-{L}_{amo})/w/2$$

2 $${R}_{proj,c}={R}_{s,proj}\ast ({L}_{line}-{L}_{amo})/w/2$$

3 $${R}_{amo}={R}_{s,amo}\ast {L}_{amo}/w$$

4 $${R}_{proj,a}={R}_{s,proj}\ast {L}_{amo}/w$$

Here, R_s,cyst_ denotes the sheet resistance of the crystalline phase change material, R_s,amo_ the sheet resistance of the melt-quenched amorphous phase change material, and R_s,proj_ the sheet resistance of the projection material. Additional circuit elements are the contact resistances of the metal electrodes to the projection and phase change material and the interface resistance between the phase change material and projection layer. These resistors may be non-negligible if a Schottky barrier forms at the material interface or simply if the contact is confined to nanoscopic dimensions smaller than the transfer length^26^. The transfer of current from one material to another is not spread evenly along the contact length, but mainly confined to the transfer length, which is defined by the contact resistivity and sheet resistance. The formation of a Schottky barrier has been reported for example at the interface between amorphous Ge_2_Sb_2_Te_5_ and TiN^27^. Based on these resistance values, it is possible to determine the overall resistance of the device (see Supplementary Note 1).

To gain insights into the state dependence and temporal evolution of the device resistance we employ the device model. We compare the two most extreme scenarios of zero and infinite interface resistance. The material parameters assumed for this calculation are shown in Table 1. The resistance values were chosen such that the typical requirements for CMOS integration in large scale crossbars are satisfied. For these purposes, the device SET resistance should be on the order of several tens of kΩ and the maximum reset resistances close to 1 MΩ or larger in order to minimize the ‘IR’ voltage drop on the interconnects which would reduce the computational precision when performing in-memory analog computing^28^. Here, the contact resistances to the metal electrodes are disregarded in order to focus on the effect of the interface resistance between phase change material and projection layer. The contact resistances will be included in the next section for the experimental validation of the model. The drift coefficient associated with the amorphous phase change material, *v*_R_, is assumed to be 0.1.

**Table 1:** Material parameters used in the device model study (Fig. 1): R_s_ denotes the material sheet resistances. In this table, the contact resistance to the electrodes is assumed to be zero to study only the effect of the interface resistance between phase change material and projection layer.

R_s,cryst_ [kΩ/sq]	20
R_s,amo_ [kΩ/sq]	5000
R_s,proj_ [kΩ/sq]	500
R_ele—PCM_ [Ω]	0
R_ele—proj_ [Ω]	0
*v*_R_	0.1
L_line_ [nm]	100
w [nm] (line width)	50

First, we study the state dependence of resistance. In the case of no interface resistance (R_int_ = 0 Ω) the device comprises three serial elements of parallel resistors (see Fig. 1b top). Two are crystalline regions parallel to the projection layer and one is the amorphous region parallel to the projection layer. All resistors in this equivalent circuit scale linearly with the length of the amorphous region in the line-cell. Thus, the device reset resistance scales linearly with the amorphous fraction of the device L_amo_/L_line_ (blue trace in Fig. 1c). In the case of infinite interface resistance (R_int_ = Inf Ω) the device comprises of two parallel resistors (Fig. 1b bottom). The entire projection layer is parallel to the phase change layer due to the infinite interface resistance. In other words, a fixed resistor (the projection layer) is connected in parallel to a resistor that changes with the amorphous fraction (the phase change layer). Consequently, the device resistance no longer scales linearly with the amorphous fraction as previously observed (red trace Fig. 1c).

Next, we study the temporal evolution of device resistance. By simulating the device model, it can be inferred that drift in a projected PCM device deviates from the standard power law for resistance drift ($R={R}_{0}\ast {(t/{t}_{0})}^{{{\rm{\nu }}}_{R}}$) (Fig. 1d) irrespective of the amorphous fraction or the interface resistance. The reason is that as the resistance of the amorphous phase increases with time, the current flowing through the amorphous plug decreases, whereas the current flowing through the element R_proj,a_ remains constant (assuming a negligible change of the voltage drop over the amorphous segment). Hence over time, the drift suppression in a projected device becomes stronger and the effective drift coefficient time dependent. The temporal dependence of the drift coefficient is however, influenced by the interface resistance.

In the zero-interface resistance scenario, the ratio between amorphous volume (R_amo_) and parallel projection segment (R_proj,a_) is independent of L_amo_, since both elements scale with L_amo_ (Eqs. 3 & 4). The current fraction flowing in the amorphous material, affected by drift, is independent of L_amo_. For small amorphous volumes, when the resistance of the crystalline fraction is non-negligible compared to R_amo_ | |R_proj,a_, the drift coefficient is reduced further. The more the overall device current is determined by the crystalline segment in series with the drifting circuit element (R_amo_ | | R_proj,a_), the less apparent is the effect of drift on the overall device current. Thus, device states with smaller amorphous fraction exhibit a slightly lower effective drift coefficient (blue trace in Fig. 1e). A more detailed explanation is provided in Supplementary Note 2.

On the other hand, in a device with infinite interface resistance, projection layer and phase change layer are electrically separated. The projecting segment here is constant (R_proj_ = R_proj,a_ + R_proj,c_ = 1 MΩ). Instead of bypassing only the amorphous volume, the read current must bypass the entire phase change layer. Compared to the zero interface-resistance scenario the fraction of read current flowing in the amorphous volume will always be larger. Moreover, the ratio of current flowing in the projection layer and the phase change layer will be strongly state dependent. The smaller the amorphous region, the larger the fraction of read current passing through it. Accordingly, smaller amorphous regions lead to a larger effective drift coefficient and drift suppression is worse than in the zero interface-resistance scenario (red trace in Fig. 1e).

The device model we propose is an approximate picture of the real device. Our goal is to provide an easily comprehensible and tractable model that is capable to capture key device metrics. There are two potential shortcomings of the model.

First, the field dependence of the amorphous phase’s transport characteristics is neglected. Hence, the results of our study are valid only in case the device is read in the ohmic regime. In the antimony device studied in this work, we measured an ohmic regime for read voltages smaller than ~0.15 V. For doped Ge_2_Sb_2_Te_5_ mushroom cells, ohmic transport has been reported for read voltages smaller than ~0.2 V^29^. We assume that it is usually feasible to perform the device read in the ohmic regime.

Second, we define a localized interface resistor between phase change material and projection layer at the boundary of the crystalline and amorphous phase change material. In reality, the device current will flow from the crystalline material to the projection layer in an extended range around the boundary of crystalline and amorphous phase change material. For metal to semiconductor contacts, this range is defined by the transfer length L_t_ = (ρ_c_/R_sh_)^0.5^, where ρ_c_ is the contact resistivity and R_sh_ is the sheet resistance of the material from which the current is flowing to another material^26^. Accordingly, the material sheet resistances and the interface resistivity determine how localized the current flow from one material to another is. Extended FEM simulations confirm that the effect of a distributed interface resistivity can be appropriately described by a localized effective interface resistance located at the boundary between amorphous and crystalline phase change material (Supplementary Note 3). The reset resistance as a function of the amorphous fraction and the drift coefficient as a function of the amorphous fraction, obtained from FEM simulations and our model, match (Supplementary Fig. S4). In the next sections, we verify the predictions from the device model shown here experimentally on nanoscale antimony PCM line-cells.

## Experimental validation of the device model

### Projected antimony line-cells

In our experimental study, we characterize unprojected and projected line-cells based on pure antimony (Sb). Recently, we have shown that antimony can be used in a PCM device when it is confined in a nanoscale volume, holding the potential for ultimate scalability and improved cyclability^30^. A device that combines the improved characteristics due to projection with a single elemental phase change material could be of great interest for next-generation PCM devices. The electrical characterization of the devices is performed at an ambient temperature of 200 K, to exclude potential recrystallization effects in our measurements.

**Figure 2:**
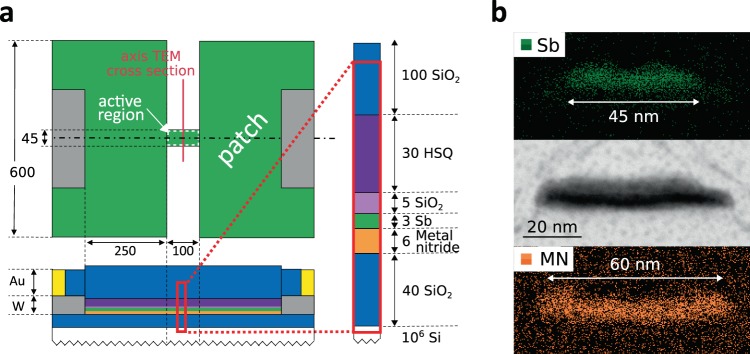
Projected antimony line-cell (**a**) Sketch of the device geometry in top and cross-sectional view; dimensions given in nanometers. HSQ: hydrogen silsesquioxane. (**b**) STEM and EDX analysis of the active region cross section along the axis marked in a. Top and Bottom panels show an EDX-map for antimony (green) and the metal nitride (orange). These images are used to get an estimate of the line width. The central panel is a bright field STEM image.

In the projected device, a 3 nm thick sputter deposited Sb layer is patterned to the structure shown in Fig. 2a. To observe and confirm the microstructure of the active region, a TEM lamella cross section was prepared and investigated by Scanning Transmission Electron Microscopy (STEM) and energy dispersive X-ray spectroscopy (EDX) (Fig. 2b). The phase change material is confined to a 100 nm long and 45 nm wide line in the active region of the device. The material is melt-quenched and recrystallized in this area. Underneath the phase change line is a 60 nm wide and 6 nm thick stripe of a metal nitride, used as projection material. The different widths of projection and phase change lines measured from the EDX elemental map (in a STEM cross-section perpendicular to the line) result from the mismatch of the etching rates of the phase change and projection layer during the fabrication process. The width of the phase change material is reduced by 15 nm due to over-etching. The unprojected device has an identical structure but without the metal nitride layer. For this device, we measure a reduction of line width due to over-etching of approximately 8 nm.

To quantify the resistive elements of the equivalent circuit in our model for these devices, we characterized the unprojected Sb line-cells and thin-film reference structures of the metal nitride. We obtained the sheet resistance (R_s_) of all individual materials (Table 2). The contact resistance between W-Sb and W-Proj was measured on four-point probe structures. Our model describes the device as a confined line in direct contact with the metal electrodes (Fig. 1a). In the real device the active region of the phase change material, the confined line, is extended to large patches contacted with W (Fig. 2a). Only in the active region of the device antimony is melt-quenched and recrystallized. The patch area does not take part in the switching process. Thus, the patch area is considered as a resistor in series with the active region. In our model, the contact resistances of Sb and projection material to W, R_W−Sb_ and R_W−proj_, include the resistance of the material interface and the resistance of the patch area (see Supplementary Fig. S10) [R_W−Sb_ = R_cont(W−Sb)_ + R_patch(Sb)_ & R_W−proj_ = R_cont(W−proj)_ + R_patch(proj)_]. Details of the experimental procedures are provided in Supplementary Note 4.

**Table 2:** Experimentally obtained model input parameters: The table summarizes the material sheet resistances in kΩ/sq and contact resistances to the W electrode in kΩ at an ambient temperature of 200 K.

R_s,proj_ [kΩ/sq]	21.8
R_s,cryst_ − Sb [kΩ/sq]	1.26
R_s,amo_ − Sb [kΩ/sq]	410 ± 60
R_W−Sb_ [kΩ]	1.6
R_W−proj_ [kΩ]	78 to 202

**Figure 3:**
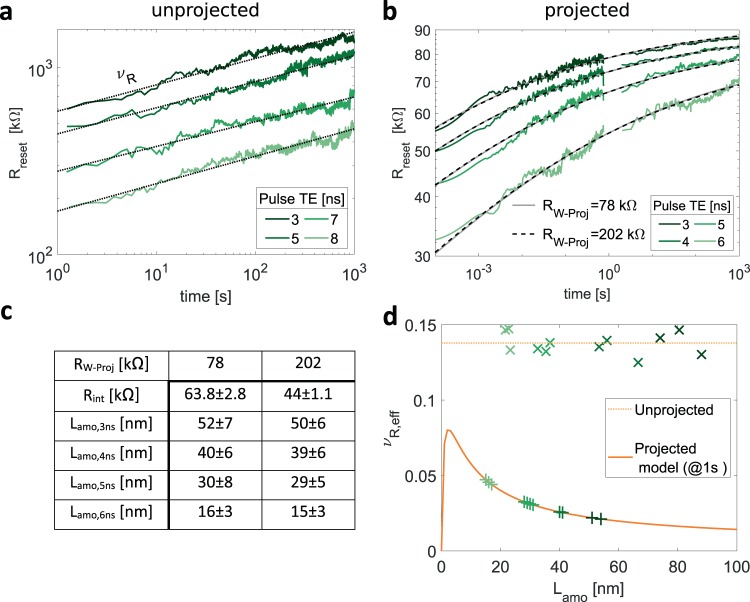
Drift measurements of unprojected and projected antimony line-cells. Different device states were obtained by varying the reset pulse trailing edge. (**a**) Four resistance drift measurements of the unprojected device. TE denotes the trailing edge. (**b**) Projected line-cell resistance drift. Resistance measurements up to 700 ms were obtained with an oscilloscope (details in Supplementary Note 5). Measurements from two seconds onwards were obtained with a source meter unit. The experimental data is fitted to the device model presented in Fig. 1a. For both the upper and lower bound of R_W−proj_ (78 kΩ and 202 kΩ) the model describes the experimental data (black and grey line). The obtained fitting parameters are summarized in the table (**c**). Those are the interface resistance and an individual amorphous length for each reset state. The error margin is the deviation of fitting parameters if the system is solved for the upper and lower error margin of R_s,amo_ – Sb (Table 2). (**d**) Effective drift coefficient as a function of the amorphous length. The drift coefficient of the unprojected cell is state independent. The amorphous length of each reset state is marked by crosses. It is calculated from the device resistance measured 1 second after RESET, for an amorphous sheet resistance of 410 kΩ/sq. In total 12 drift measurements were performed (Supplementary Fig. S14). The state dependent drift coefficient of the projected cell is calculated from the model. Crosses mark the amorphous length of R(t) measurements used for the model fit. On the projected device 16 drift measurements were performed (Supplementary Fig. S15).

The sheet resistance of the amorphous state corresponds to the melt-quenched state one second after device RESET. The contact resistance W to projection material was measured on macroscopic reference structures and extrapolated to the nanoscopic contact area in the device. Accordingly, R_W−proj_ is estimated with lower and upper bounds of 78 kΩ and 202 kΩ, respectively. The errors on the other parameters are negligible, and thus excluded in the analysis. Details on the experimental procedure to obtain these parameters are summarized in Supplementary Note 4. The material parameters summarized here are used to fit the experimentally obtained data to the device model (Fig. 3).

### Resistance drift

To examine if our device model is able to capture the drift characteristics of the projected antimony line-cell, we first need to characterize the resistance drift of the unprojected device. The line-cell is programmed to different reset states at a constant programming current of 610 µA by varying the reset pulse trailing edge between 3, 5, 7 and 8 ns. With increasing pulse trailing edge (duration of melt-quenching process), the reset resistance decreases, because a larger amount of material crystallizes during melt-quenching. After programming, the evolution of the resistance as a function of time is measured over 1000 s and fitted to the standard drift equation $R={R}_{0}\ast {(t/{t}_{0})}^{{{\rm{\nu }}}_{R}}$ (Fig. 3a). We measure a drift coefficient of 0.14 ± 0.01 for four different intermediate states and find no apparent dependence of the drift coefficient on the pulse trailing edge. Hence, the drift coefficient is independent of the amorphous length. It is noted that the drift coefficient measured here at 200 K is larger than the drift coefficient of 0.1 ± 0.02 reported previously for an ambient temperature of 100 K^30^. Measurements on a single line-cell at 100 K and 200 K indeed confirm that the drift coefficient changes from 0.1 to 0.14 (Supplementary Fig. S13).

We now return to the experimental characterization of the projected device. The model predicts a deviation of the resistance drift from the standard relation. To validate this, we experimentally measure the time dependent resistance of four different reset states of a projected Sb line-cell (Fig. 3b). In accordance with the model, the experimentally measured logarithmic evolution of the device resistance exhibits a clear curvature. Additionally, the separation between the resistance states decreases with time, indicating a non-zero interface resistance between the Sb and projection layer.

In order to verify if the device model can capture the measured R(t) data, we attempt to fit it to the model. Having predefined all experimentally directly accessible input parameters for our model (Table 2) and the drift coefficient of antimony, two fit parameters are left to describe the R(t) data of an individual reset state. Those are the interface resistance between antimony and metal nitride and the amorphous length of the reset state (Fig. 1a). We perform a collective fit of the R(t) traces measured for four different reset states. The model is able to describe these traces using a single value of interface resistance and four different amorphous lengths. Of the experimentally determined model input parameters, the contact resistance R_W−proj_ was estimated with the greatest uncertainty. For both the lower and upper bounds of R_W−proj_, the fit captures the experimental data well. The amorphous length obtained from the fit is approximately independent of R_W−proj_ (Fig. 3c). Hence, in the model, the two parameters R_W−proj_ and R_int_ compensate each other. These two parameters determine the fraction of read current that is injected into the projection layer and thus not changed by drift. The model fit also reveals upper and lower bounds for the last unknown variable in our model, the interface resistance. With all model parameters at hand, we calculate the state dependent effective drift coefficient of the projected line-cell one second after resetting the device (Fig. 3d). Depending on the length of the amorphous volume, the drift coefficient is suppressed by a factor of two (for L_amo_ = 2 nm) up to a factor of 10 (for L_amo_ = 100 nm) compared to the unprojected device.

### State dependence of the reset resistance

**Figure 4:**
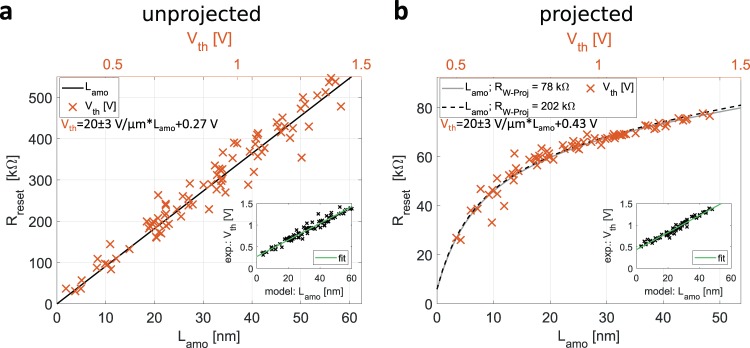
State dependence of the reset resistance (**a**) unprojected line-cell, (**b**) projected line-cell. State dependence refers to the dependence of the device resistance on the phase configuration of the phase change material i.e. the amorphous length. Black lines depict the expected scaling of reset resistance with the amorphous length. The scaling of reset resistance with amorphous length of the projected line-cell is calculated from the device model with the fit parameters summarized in Table 2 and Fig. 3c. Crosses mark the experimentally obtained data R_reset_ vs V_th_. To match experimental data (top X-axis) and calculated data (bottom X-axis) the axes are scaled with the linear function noted in the left corner of the graph. The insets show measured V_th_ against L_amo_ for identical R_reset_ values. The linear fit of this data (green line) gives the function used to scale the top and bottom x-axis.

To investigate the state dependence of the reset resistance, we first perform experiments on an unprojected line-cell where the model predicts that the reset resistance scales linearly with the amorphous length as shown in Fig. 4a. We calculate the reset resistance for a 52 nm wide line-cell as R_reset_(L_amo_) = [R_s,amo_ * L_amo_ + R_s,cryst_ * (L_line_ − L_amo_)]/w, where w denotes the device width, R_s,amo_ the amorphous sheet resistance one second after device RESET, R_s,cryst_ the crystalline sheet resistance, L_amo_ the amorphous length and L_line_ the device length. However, to verify this model, we need an estimate of the amorphous length. The threshold switching behavior in amorphous phase change materials provides us an ingenious way to obtain an indirect experimental measure of the amorphous length. The threshold switching voltage (V_th_) is assumed to be linked to the amorphous length (L_amo_) by the equation, V_th_ = E_th_ * L_amo_ + V_offset_^31^ where E_th_ is the threshold field and V_offset_ an offset voltage. The device is reset to different states by varying the programming power. The values of R_reset_ one second after RESET and V_th_ are measured. From Fig. 4a, it can be seen that R_reset_ scales linearly with V_th_ and hence with L_amo_ as predicted by the model. This experiment also enables us to establish the fit function relating L_amo_ to V_th_, given by V_th_ = 20 ± 3 V/µm * L_amo_ + 0.27 V (inset in Fig. 4a). The error margin is defined by the propagated error of R_s,amo_, which results in an error on the model parameters R_int_ and L_amo_ (Fig. 3c andSupplementary Note 4).

Unlike the unprojected devices, for the projected line-cells, based on the model parameters obtained from the previous section, R_reset_ is expected to be a non-linear function of L_amo_ (Fig. 4b). To experimentally verify this, we make the assumption that the threshold voltage is still a linear function of the amorphous length. Experimental measurements of R_reset_ and V_th_ are obtained on a projected line-cell as shown in Fig. 4b. Combining the model data R_reset_(L_amo_) with the experimental data R_reset_(V_th_) facilitates the mapping of an experimentally measured V_th_ to the corresponding L_amo_ even for the unprojected devices. Fitting L_amo_(V_th_) linearly (inset in Fig. 4b), we obtain the identical E_th_ as from characterizing the unprojected device. The predicted state-dependence of reset resistance is corroborated. The curvature of R_reset_(V_th_) matches the model prediction for R_reset_(L_amo_). Additional information of the threshold switching dynamics and threshold field are provided in Supplementary Note 6 & 7.

## Model Exploration

Having validated the proposed device model experimentally, we assess the implications for device applications and the selection of materials for a projected memory device. Resistance drift is an unfavorable device characteristic since it leads to a loss of information as device states are shifting to different levels. However, if all devices programmed in a chip exhibit the same drift coefficient, it is possible to account for drift and compensate it at a global scale. Thus, the state dependence of device drift introduced by a large interface resistance is unfavorable even though the projection layer may reduce the absolute device drift. A non-linear scaling of reset resistance with amorphous length may also introduce challenges in a device application. The resistance of low reset states with small amorphous lengths changes significantly with the size of the amorphous volume. Thus, it becomes more challenging to program device target states in the low resistance range. Consequently, only a fraction of the possible device states may be exploited. The resistance dynamic range R_off_/R_on_ and number of resistance states that can be distinguished reliably decrease.

It can also be seen that even though finite interface resistances pose a challenge to the concept of the projected PCM, within certain bounds they can be tolerated without detrimental effects to the device characteristics. We define three constraints for the device metrics to identify those bounds. First, the maximum drift coefficient should be smaller than 0.01. Second, to restrict drift variability, the resistance separation between the most and least drifting device state must not change by more than 5% in the time window 1 s to 10^4^s. Third, to enforce an approximately linear scaling of device resistance with the amorphous length, the R(L_amo_) curve should not deviate by more than 20% from a linear interpolation between R_DUT,min_ an R_DUT,max_. A more detailed explanation of the constraints is provided in Supplementary Note 8.

**Figure 5:**
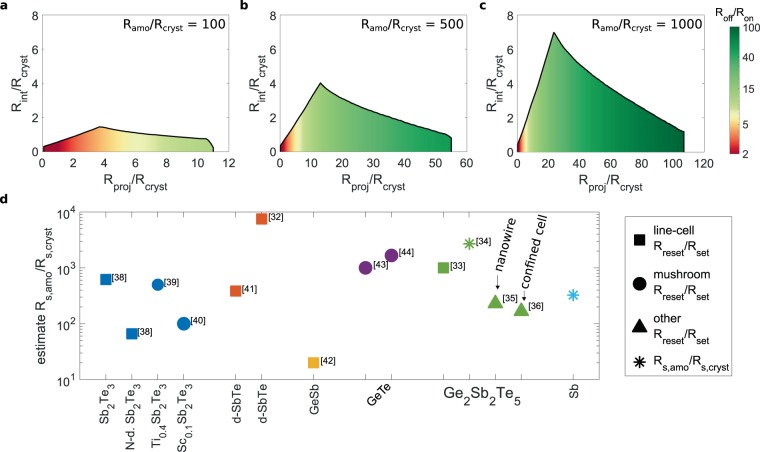
Guidelines for device optimization. Target specifications for future device generations are a drift coefficient smaller than 0.01, a minor state dependence of the drift coefficient and a close to linear scaling of the resistance with the amorphous length. For defined resistance ratios of amorphous to crystalline sheet resistance (**a–c**) the colored area of the graphs marks feasible projection layer sheet resistances and interface resistances. The color gradient encodes the device OFF/ON ratio. (**d**) Estimate of the melt-quenched amorphous to crystalline state resistance ratio of different phase change materials. The estimate is obtained from device programming curves. Marker shapes encode the device geometry, compounds of comparable composition are clustered and share a color. ref. ^32–36,38–44^.

Since these device characteristics are merely determined by the ratios of the material sheet resistances, we can obtain a generic solution as a guideline for material selection. Here, we study how the maximum interface resistance that can be tolerated to fulfil the aforementioned constraints changes with R_s,proj_ for three different resistance ratios of amorphous to crystalline phase change material R_s,amo_/R_s,cryst_ (Fig. 5a–c). The curves of the maximum interface resistance can be split into two regimes to the left and right of the maximum. In regime one, at small projection layer resistances, the maximum interface resistance that can be tolerated increases steeply. The projection layer sheet resistance is much smaller than the amorphous sheet resistance. In this case, the requirement to limit the curvature of R(L_amo_) determines the interface resistance. A drift coefficient smaller than 0.01 could also be achieved with large interface resistances. In the second regime, the maximum interface resistance gradually decreases. The limiting constraint is the requirement of a maximum drift coefficient smaller than 0.01. As the projection layer sheet resistance increases and gets closer to the amorphous sheet resistance, the projection must become “better” to suppress the drift sufficiently. A better projection is enabled by a smaller interface resistance. Eventually, the projection layer resistance becomes too large to suppress the drift to 0.01. With increasing ratio of R_amo_/R_cryst_ (Fig. 5a–c) the maximum shifts to larger values of R_proj_/R_cryst_ and R_int_/R_cryst_. Furthermore, the largest projection layer resistance for which the drift coefficient is smaller than 0.01 increases. Consequently, larger device OFF/ON ratios can be realized (color coded). Large OFF/ON ratios (green) are highly desirable to either widen the separation of device states, which would make it more fault tolerant, or else to increase the number of states that can be encoded. To fabricate a device with a large OFF/ON ratio a phase change material with large R_amo_/R_cryst_ is preferable. A projection material and phase change material combination that simply maximizes the interface resistance that can be tolerated is not advantageous. Instead a compromise between device OFF/ON ratio and acceptable interface resistance must be made.

To assess the feasibility of different phase change materials for a projected device, we need to know the resistance ratio of the melt-quenched amorphous phase and the crystalline phase. Whilst numerous papers report the resistivity of the as deposited amorphous phase only limited data has been reported for the melt-quenched amorphous phase, the material state of interest in device applications. We use the reset to set resistance ratio reported for device programming curves to estimate the resistance ratio of melt-quenched amorphous and crystalline state (Fig. 5d). The maximum reset state created in the programming curves we analyzed appears to be a saturation level, suggesting the size of the amorphous volume has reached a maximum. We note that a saturation of the programming curve does not necessarily correspond to a fully amorphous device. Thus, our analysis may underestimate the materials resistance contrast. The studies of doped-SbTe^32^, and Ge_2_Te_2_Sb_5_^33,34^ show TEM figures of an amorphized device, suggesting the complete device volume has been amorphized. These two materials and GeTe, all exhibiting resistance ratios larger than 1000, are the most suitable candidates for a projected memory device. However, device studies of highly confined Ge_2_Sb_2_Te_5_^35,36^ report a significantly reduced resistance ratio. The programming data reported for N-doped Sb_2_Te_3_, Sc_0.1_Sb_2_Te_3_ and GeSb suggests these materials are not well suited for a projected device. Due to their small resistance ratios (<100) it may be challenging to apply them for a drift and noise resilient multi-level memory device. For materials with resistance ratios <500 a careful optimization of the projection material, especially the interface resistance, will be required. Larger crystalline sheet resistances will allow absolute larger interface resistances to be tolerated.

## Conclusion

Projected PCM is arguably the most promising approach towards realizing precise in-memory computing using PCM devices. We have studied the key attributes of a projected phase change memory device, namely, the state dependence and the temporal evolution of resistance. We developed a device model that can describe quantitatively the experimental data obtained from projected PCM devices based on antimony as the phase change material. One of the key insights from the model is the importance of the interface resistance between the projection layer and the phase change material. The magnitude of the interface resistance that can be tolerated in a device depends on the sheet resistance ratios of the projection material, and the crystalline state and amorphous states of the phase change material. With the improved understanding of the device characteristics, we developed a guideline for device engineering, which can be universally applied for projected memory device concepts, as well as in other applications where devices are comprised of heterostructures. The presented work is a significant step towards the directed design of large arrays of projected PCM devices for neuromorphic and in-memory computing.

## Methods

### Device fabrication

The devices were fabricated on a silicon substrate with a 40 nm thermally grown SiO_2_ dielectric top layer. For the unprojected line-cell, a layer stack of 3 nm Sb and 5 nm SiO_2_ was sputter deposited. For the projected line-cell, a layer stack of 6 nm amorphous metal nitride, 3 nm Sb and 5 nm SiO_2_ was deposited. The stacks were sputter deposited without breaking the vacuum. The line-cell geometry was defined by e-beam lithography with hydrogen silsesquioxane resist and transferred to the layer stack by ion-milling. The structure was passivated with 18 nm sputter deposited SiO_2_ immediately after pattern transfer. To electrically contact the device structure, the capping was locally opened in a second e-beam lithography step and Tungsten was sputter deposited. The Tungsten was patterned in another e-beam lithography step followed by ion-milling to extended electrodes. A titanium resistor, also defined by e-beam lithography, was added in series to the Sb-device (projected device: 2.8 kΩ; unprojected device: 3.5 kΩ) to limit the current during threshold-switching. The chip was encapsulated with an 80 nm sputter deposited SiO_2_ layer. Finally, gold probe pads (200 nm; sputter deposited) were connected to the tungsten.

### Structural characterization

To analyze the structural quality of the projected antimony line-cell and check the chemical species present, a cross-sectional lamella was investigated. It was prepared using a FEI Helios Nanolab 450 S focused ion beam and characterized by scanning transmission electron microscopy (STEM). The bright field images were acquired using a double spherical aberration-corrected JEOL JEM-ARM200F microscope operated at 200 kV and the chemical composition was investigated by energy dispersive Xray spectroscopy (EDX) using a liquid-nitrogen free silicon drift detector.

### Electrical characterization

The electrical measurements of the line-cells were performed in a cryogenic probing station (JANIS ST-500-2-UHT) at an ambient temperature of 200 K. The temperature was measured with Lake Shore Si DT-670B-CU-HT diodes at four positions in the chamber. A Lake Shore Model 336 automatic temperature controller and two heaters at the sample holder and chamber radiation shield controlled the temperature with an accuracy of 0.5 K. The chamber was evacuated to 10^−5^ mbar to minimize heat exchange via convection and avoid water condensation. Devices were electrically contacted with a high-frequency Cascade Microtech Dual-Z Probe. The probe was thermally connected to the sample holder with cooling braids.

DC measurements of the device state were performed with a Keithley 2600 System SourceMeter. AC signals were applied to the device with an Agilent 81150 A pulse function arbitrary generator. A Tektronix oscilloscope (DPO5104) recorded the voltage pulses applied to and transmitted by the device. Switching between the circuit for DC and AC measurements was done with mechanical relays. A detailed description of the measurement setup can be found elsewhere^37^.

## Supplementary information

Supplementary Information.
