# Supplementary material for: State dependence and temporal evolution of resistance in projected phase change memory
Source: Sci Rep. 2020 May 19;10:8248. doi: 10.1038/s41598-020-64826-3 (PMC7237438; doi:10.1038/s41598-020-64826-3)
Supplement: Supplementary file 1 — Supplementary Information. [file 41598_2020_64826_MOESM1_ESM.docx]

**State dependence and temporal evolution of resistance in projected phase change memory**

**Supplementary Information**

Benedikt Kersting^1*^, Vladimir Ovuka^1^, Vara Prasad Jonnalagadda^1^, Marilyne Sousa^1^, Valeria Bragaglia^1^, Syed Ghazi Sarwat^1^, Manuel Le Gallo^1^, Martin Salinga^2^, Abu Sebastian^1*^

1 IBM Research – Zurich, Säumerstrasse 4, 8803 Rüschlikon, Switzerland

2 Institut für Materialphysik; Westfälische Wilhelms-Universität Münster, Wilhelm-Klemm-Straße 10, 48149 Münster, Germany

1. **Total device resistance for an arbitrary network**

To calculate the overall resistance for an arbitrary interface resistance we need to make a $\Delta$-Y transformation of our circuit. The $\Delta$ R_ele-PCM_ + R_cryst_ ; R_interface_ ; R_ele-Proj_ + R_proj,c_ is translated to the star R_1_; R_2_; R_3_ (Figure S1).


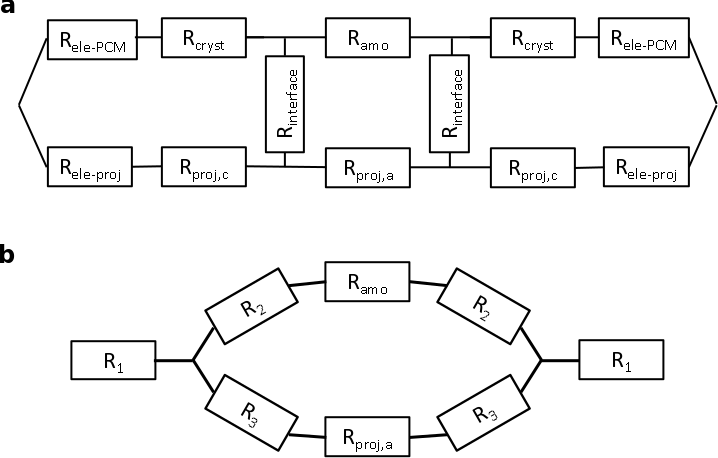


Figure S1: **Model equivalent circuit of the projected PCM cell** (a) in delta configuration and (b) in star configuration

The resistors of the equivalent circuit are described by equation 1 to 3. The total device resistance can be calculated with equation 4 as an equivalent circuit. This generalized form of R_total_ allows us to calculate the device resistance for arbitrary contact resistances to the electrodes and at the PCM to the projection layer interface.

|  | $R_{1}=\frac{\left( R_{W-Sb}+R_{cryst}/2 \right)*{(R}_{W-TaN}+R_{proj,c}/2)}{R_{W-Sb}+R_{cryst}/2+R_{W-TaN}+R_{proj,c}/2+R_{interface}}$ | (1) |
| --- | --- | --- |
|  | $R_{2}=\frac{\left( R_{W-Sb}+R_{cryst}/2 \right)*R_{interface}}{R_{W-Sb}+R_{cryst}/2+R_{W-TaN}+R_{proj,c}/2+R_{interface}}$ | (2) |
|  | $R_{3}=\frac{\left( R_{W-TaN}+R_{proj,c}/2 \right)*R_{interface}}{R_{W-Sb}+R_{cryst}/2+R_{W-TaN}+R_{proj,c}/2+R_{interface}}$ | (3) |
|  | $R_{total}=2*R_{1}+\frac{\left( 2R_{2}+R_{amo} \right)*\left( 2R_{3}+R_{proj,a} \right)}{2R_{2}+R_{amo}+2R_{3}+R_{proj,a}}$ | (4) |

1. **Projected line-cell drift coefficient – R_int_ = 0**

In a projected line-cell with no interface-resistance between projection and phase change material, the ratio of current flowing in the amorphous volume to current flowing in the parallel projection element is independent of the amorphous length. Without any other circuit elements involved, the effective drift coefficient $\nu$_R,eff;alone_ would be independent of the amorphous length (Figure S2a). The additional circuit element in a real device is the crystalline phase change material that is electrically connected in series to the drifting circuit element R_drift_ = R_amo_||R_proj_. We define R_drift1_(t_1_), R_drift2_(t_2_) and R_cryst_ = x*R_drift1_, where R_drift1_ is the resistance of the drifting circuit element at t_1_ and R_drift2_ at t_2_. X denotes the resistance ratio of the crystalline phase change material and the drifting element at t_1_. This ratio is a function of the amorphous fraction (). The effective drift coefficient of the drifting element alone is defined in Equation (5), and the effective drift coefficient of the drifting element in series with a crystalline element $\nu$_R,eff;ser_ in Equation (6). R_cryst_ is an offset, which increases the total device resistance. The drift coefficient is defined by the resistance ratio between two time instances t_1_ and t_2_. The larger the ratio R_cryst_/R­_drift_ the smaller the effective drift coefficient. The absolute change of device resistance is the same in Equation (5) and Equation (6), but the resistance offset results in a smaller effective drift coefficient. If t_2_ approaches t_1_, R_drift2_ approaches R_drift1_. The limit as R_drift2_ approaches R_drift1_ allows us to obtain the ratio of $\nu$_R,eff;ser_/$\nu$_R,eff;alone_ (Equation (7)). The larger x, which is defined as the ratio R_cryst_/R­_drift_ (i.e. the smaller the amorphous fraction), the smaller is the effective drift coefficient.

|  | $\nu_{R,eff;alone}=\frac{log\left[ \frac{R_{drift1}}{R_{drift2}} \right]}{log\left[ \frac{t_{1}}{t_{2}} \right]}$ | (5) |
| --- | --- | --- |
|  | $\nu_{R,eff;ser}= \frac{log\left[ \frac{\left( R_{drift1}+R_{cryst} \right)}{\left( R_{drift2}+R_{cryst} \right)} \right]}{log\left[ \frac{t_{1}}{t_{2}} \right]}=\frac{log\left[ \frac{\left( R_{drift1}+x*R_{drift1} \right)}{\left( R_{drift2}+x*R_{drift1} \right)} \right]}{log\left[ \frac{t_{1}}{t_{2}} \right]}$ | (6) |
|  | $\lim_{R_{drift2}\to R_{drift1}}(\frac{\nu_{R,eff ser}}{\nu_{R,eff alone}}) = \frac{1}{1+x}$ | (7) |


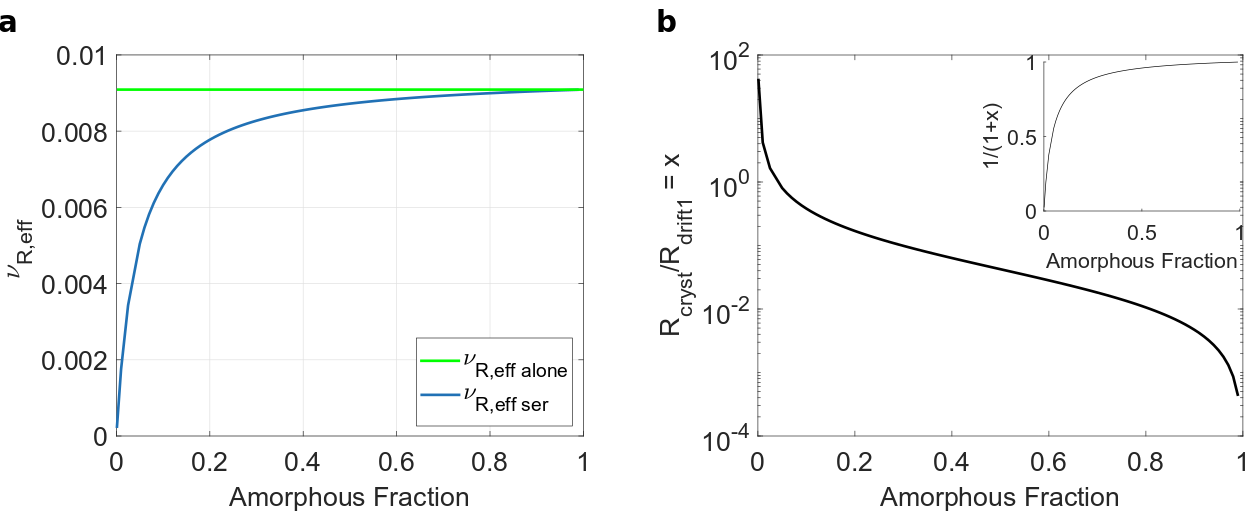


Figure S2: **Impact of R_cryst_ in series with R_drift_ = R_amo_||R_proj,a_ on the drift coefficient**, 1 s after RESET) (a) Drift coefficient obtained from the equivalent circuit device model. $\nu_{R,eff alone}$ denotes the drift coefficient calculated if R_cryst­_ is neglected, $\nu_{R,eff ser}$ denotes the drift coefficient obtained for the complete equivalent circuit. In the equivalent circuit model, the drift is calculated from the resistance change between t_1_ = 1s-1µs and t_2_ = 1s+1µs (b) Ratio of crystalline resistance to resistance of the drifting element. The inset shows the drift suppression calculated from (7).

1. **Comparison of the device model to FEM simulations**

In this note, we compare our device model with the localized interface resistance to an FEM simulation for which we define an interface resistivity between phase change material and projection layer.

*The FEM model geometry*

For the FEM simulation, we create a 2D model of the device (Figure S3). It resembles our device model presented in Figure 1a. The line-cell is 100 nm long. The projection layer is 6 nm thick and the phase change material 3 nm thick. We define a one nm spacing for the mesh grid points in the phase change material and projection material. For the interface between projection layer and phase change material, we define an interface resistivity $\rho$_interface_ in Ω*m^2^.


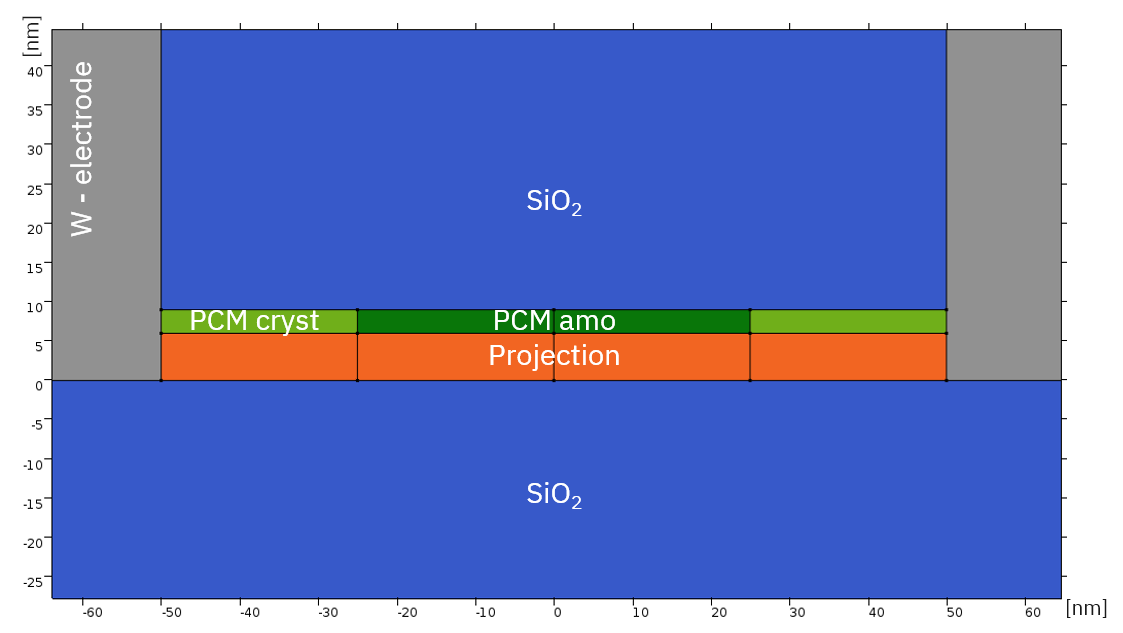


Figure S3: **FEM model geometry**. The materials assigned to the geometry elements are color coded. For the interface between PCM and projection material an interface resistivity is defined in the model. The amorphous length in this figure is 50 nm.

*Reset resistance and drift coefficient*

We compare the two key metrics studied in this paper for model and FEM simulation. Those are the changes of reset resistance and drift coefficient as a function of the amorphous fraction. To make the results comparable, we assign the same material and geometry parameters to all elements of the two models (Table S1). The material resistivities and thicknesses assigned in the FEM simulation result in the sheet resistance used in our device model. The only difference is that we define an interface resistance in our device model, whereas an interface resistivity is used in the FEM simulation.

Table S1: Material and Geometry parameters used for the device model and the FEM simulation.

| Device Model | | FEM simulation | |
| --- | --- | --- | --- |
| R_s,cryst_ [kΩ/sq] | 20 | $\rho$_cryst_ [$\Omega$*cm] | 6*10^-3^ |
| R_s,amo_ [kΩ/sq] | 5000 | $\rho$_amo_ [$\Omega$*cm] | 1.5 |
| R_s,proj_ [kΩ/sq] | 500 | $\rho$_proj_ [$\Omega$*cm] | 0.3 |
| R_ele—PCM_ [Ω] | 0 | R_ele—PCM_ [Ω] | 0 |
| R_ele—proj_ [Ω] | 0 | R_ele—proj_ [Ω] | 0 |
| $\nu$_R_ | 0.1 | $\nu$_R_ | 0.1 |
| L_line_ [nm] | 100 | L_line_ [nm] | 100 |
| w [nm] (line width) | 50 | w [nm] | “50”; 2D simulation |
|  |  | t_PCM_ [nm] (thickness) | 3 |
|  |  | t_Proj_ [nm] (thickness) | 6 |

The FEM simulation is computed for four different interface resistivities $\rho$_int_ (inf; 10^-9^; 10^-10^; 0 [$\Omega$*m^2^]). To identify the corresponding interface resistance in our device model, we fit our device model to the reset resistance as a function of the amorphous fraction obtained in the FEM simulation. The only fitting parameter is the interface resistance R_int_ between phase change material and projection layer. For each interface resistivity we find a corresponding interface resistance that allows to reproduce the scaling of the reset resistance with amorphous fraction calculated in the FEM simulation (Figure S4 a).

We use the same parameters to compute the effective drift coefficient as a function of the amorphous fraction. The drift coefficients obtained with FEM simulation and our model match as well (Figure S4 b). These two results show that the distributed interface resistivity in a real device as well as the current flow from one layer to another over an extended range can be appropriately described by a localized effective interface resistance located at the boundary between amorphous and crystalline phase change material. We acknowledge that the link between material interface resistivity and our model interface resistance may be described by a non-trivial functionality, that has not been defined as a part of this work.


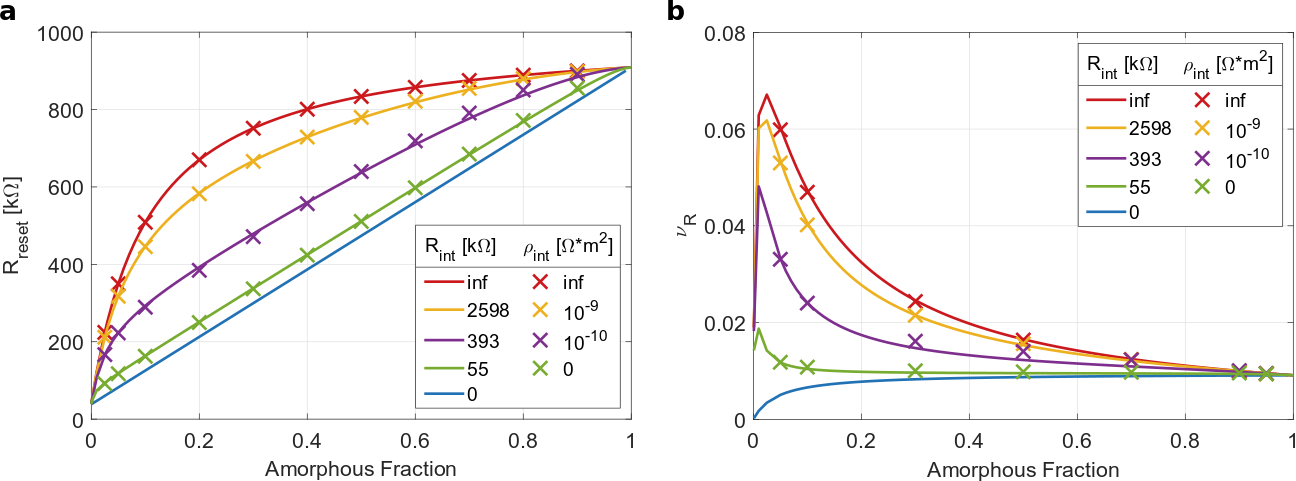


Figure S4: **Comparison FEM simulation to device model.** Data computed in the FEM simulation is marked by crosses. Data calculated with our device model is marked as a line. The legend captures the FEM interface resistivity and the corresponding interface resistance. (a) Scaling of the reset resistance with the amorphous fraction. (b) Device drift coefficient as a function of the amorphous fraction.

For the material parameters used in this simulation a zero-interface resistivity corresponds to an interface resistance of 55 kΩ. A zero-interface resistivity does not correspond to a zero-interface resistance because of the extreme confinement of current flow from phase change material to the projection layer in this scenario. This confinement results in high current densities and current crowding at the interface (Figure S5) leading to a finite effective interface resistance.


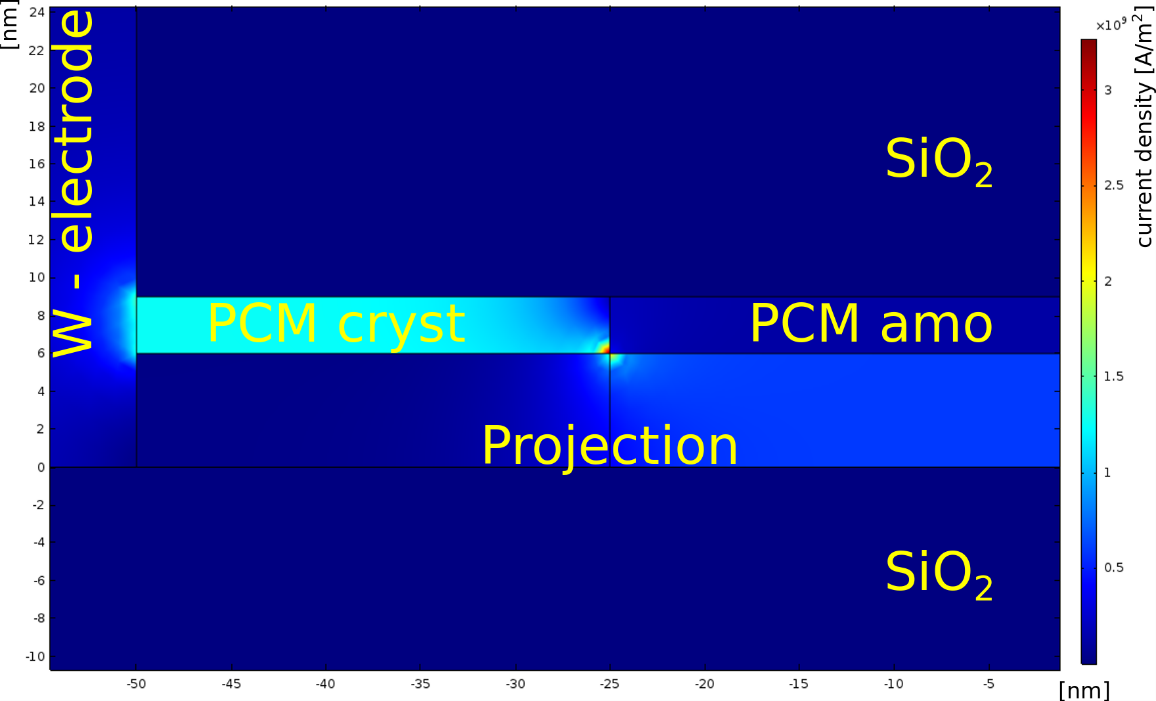


Figure S5: **Current densities in a projected device.** The interface resistivity is 0 Ω*m^2^. Shown is the device cross-section from the left electrode to the center of the device. The current density is color coded. The color bar ranges from 0 to 3.25e9 A/m^2^.

*Model Exploration*

Finally, we want to verify the guidelines for device optimization presented in Figure 5 of the manuscript. To create such a view graph, we need to obtain the reset resistance and drift coefficient as a function of the amorphous fraction for multiple projection layer resistivities (x-axis) and interface resistivities (y-axis). The view graph obtained from the FEM simulations (Figure S6 a) resembles the result calculated with our device model (Figure S6 b). Due to the comparably large computational expense of the FEM simulation, we limited the number of data points evaluated in the FEM simulation to 7 projection layer resistivities and 10 interface resistivities. Our simple device model enables us to characterize the system at 66 interface resistances and 180 projection layer sheet resistances in a fraction of the time.
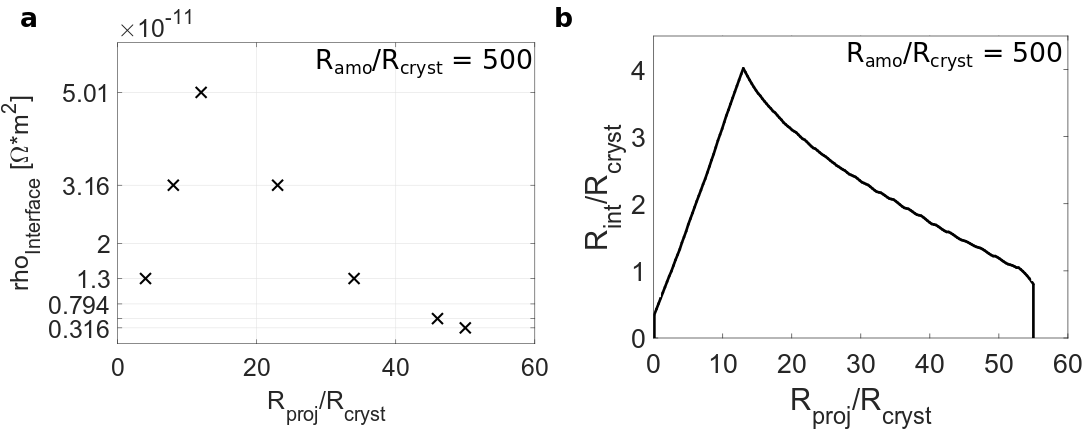


Figure S6: **Guidelines for device optimization****.** Target specifications for future device generations are a drift coefficient smaller than 0.01, a minor state dependence of the drift coefficient and a close to linear scaling of the reset resistance with the amorphous length. The area under the curve represents the parameter space that fulfills these constraints. (a) FEM simulation. Y-gridlines mark the simulated interface resistivities. The granularity had to be selected coarse due to the high computational expense of the FEM simulation. (b) device model proposed in this work.

1. **Preliminary material characterization**

*Crystalline Antimony – sheet resistance*

The sheet resistance of crystalline antimony is calculated from the pristine line-cell resistance, measured for different device geometries (Figure S7a). The slope of the resistance vs length/width of the line fit corresponds to the sheet resistance. The Y-intercept is the contact resistance. A detailed discussion follows in ‘Characterization of contact resistances to the metal electrodes. Between 200K and 300K the resistance changes by less than 2% (Figure S7b). We consider this change negligible and define in our device model a crystalline sheet resistance of 1260 Ω/sq for an ambient temperature of 200 K.


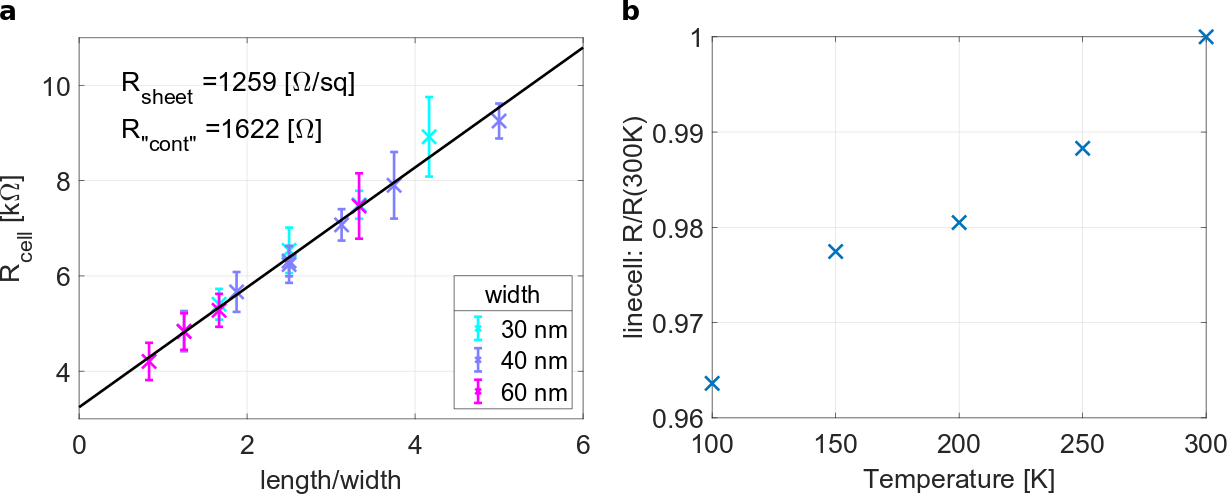


Figure S7 **Crystalline Antimony – sheet resistance** (a) Pristine line-cell resistance versus length/width of the line. On a single chip multiple cells have the same geometry. Error bars denote the standard deviation of the device resistance for an individual cell geometry. (b) Temperature dependence of the crystalline device resistance, normalized to the resistance measured at 300 K.

*Amorphous antimony – sheet resistance*

In our study we characterize a melt-quenched amorphous state. The resistance of a melt-quenched state is not to be mistaken with the resistance measured for an as deposited amorphous sample. To obtain the sheet resistance of the melt-quenched amorphous phase at an ambient temperature of 200 K we perform a series of programming experiments on unprojected line-cells. We measure reset resistance and threshold voltage of 32 nm wide devices with different line lengths from 150 nm to 50 nm (Figure S8). The measured threshold voltages and reset resistances measured for multiple reset states on the devices of different length scale linearly. A linear scaling of R_reset_ and V_th_ confirms both metrics depend linearly on the amorphous length. The same scaling behavior for devices of different length affirms the same device cross-section, as R_reset_ depends on the device cross-section. Thus, we can use the experimental data of three different devices to characterize the melt-quenched amorphous sheet resistance. To obtain an estimate of the amorphous sheet resistance, we make assumptions about the largest and smallest amorphous volume created in the device.

The first assumption is that we cannot create an amorphous length larger than the line length. Based on this assumption the maximum reset resistance measured for a particular device length enables us to define a lower bound for the amorphous sheet resistance. For the three different device lengths and their maximum reset resistance, we calculate an amorphous sheet resistance (table 1). The sheet resistance obtained from the 100 nm and 150 nm long line is 350 kΩ/sq. For the 50 nm long line, we obtain a sheet resistance of 175 kΩ/sq. We discard this value because it would require a 200 nm and 300 nm long amorphous mark to achieve the maximum reset resistance measured for the 100 nm and 150 nm long lines, respectively. Thus, we conclude the 50 nm long device cannot have been amorphized completely. The sheet resistance obtained from the other two devices suggests the largest amorphous mark in the 50 nm long device is 25 nm.

To estimate an upper bound for the amorphous sheet resistance, we make an assumption about the amorphous length of the least resistive reset state created in the 50 nm long device (74 kΩ). Here the least resistive reset state corresponds to the first change of device resistance in the programming curve. Its amorphous length must be smaller than 6.75 nm otherwise the highest resistive reset states would be larger than the device length (estimate of the lower bound for R_s,amo_). A minimum value cannot be precisely defined. We note that due to the complex temperature distribution during melt-quenching, the probability of a crystalline percolation path in the 32 nm wide cell becomes extremely high if the amorphous length gets too small. Also, it seems unlikely that the largest reset states created in the device are only a few nm long. Here we assume a minimum amorphous length of ~ 5 nm. This corresponds to an amorphous sheet resistance of 470 k kΩ/sq. In this scenario, the largest amorphous mark is 18 nm, 74 nm and 112 nm in the line-cells of 50 nm, 100 nm and 150 nm, respectively. For our model fit we assume the sheet resistance to be the central value between upper (470 kΩ/sq) and lower (350 kΩ/sq) bound, with an uncertainty ranging to both bounds R_s,amo_ = 410 kΩ/sq±60 kΩ/sq.

Table 2 **Lower bound of R_s,amo_.** The device length and maximum reset resistance are used to calculate a lower bound for the melt-quenched amorphous sheet resistance (column 3). To verify if the sheet resistance is reasonable, the amorphous length of the other two devices is calculated from their maximum reset resistance. Only sheet resistances that give an amorphous length smaller than the device length are reasonable. This constraint is not fulfilled for the 50 nm long device.

| Device Length | max (R_reset_ ) | **R_sheet_** |
| --- | --- | --- |
| 50 nm | 0.27 MΩ | 175 kΩ/sq |
| 100 nm | 1.10 MΩ | **352 kΩ/sq** |
| 150 nm | 1.66 MΩ | **354 kΩ/sq** |


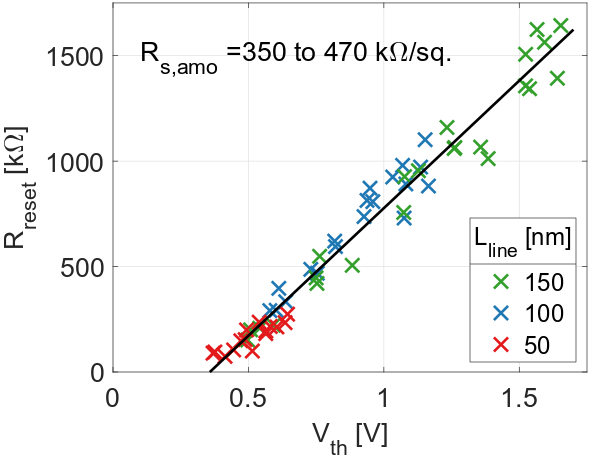


Figure S8 **Reset resistance vs Threhshold voltage** measured for devices of different length. The maximum reset resistance created for different geometries is used to estimate the sheet resistance of the melt-quenched amorphous phase. The experiment was performed at an ambient temperature of 200 K.

*Metal nitride – sheet resistance*

The metal nitride sheet resistance was measured on bar structures in four-probe configuration at room temperature (Figure S9a). To account for its temperature dependence, we measure two probe reference structures of different lengths in a temperature range from 100 K to 300 K (Figure S9b). From these measurements, we obtain the temperature dependence of the sheet resistance and contact resistance (Figure S9 c&d). Both follow an Arrhenius behavior with an activation energy of 0.024 eV and 0.056 eV, respectively. At 200K, the metal nitride has a sheet resistance of 21.8 kΩ/sq.


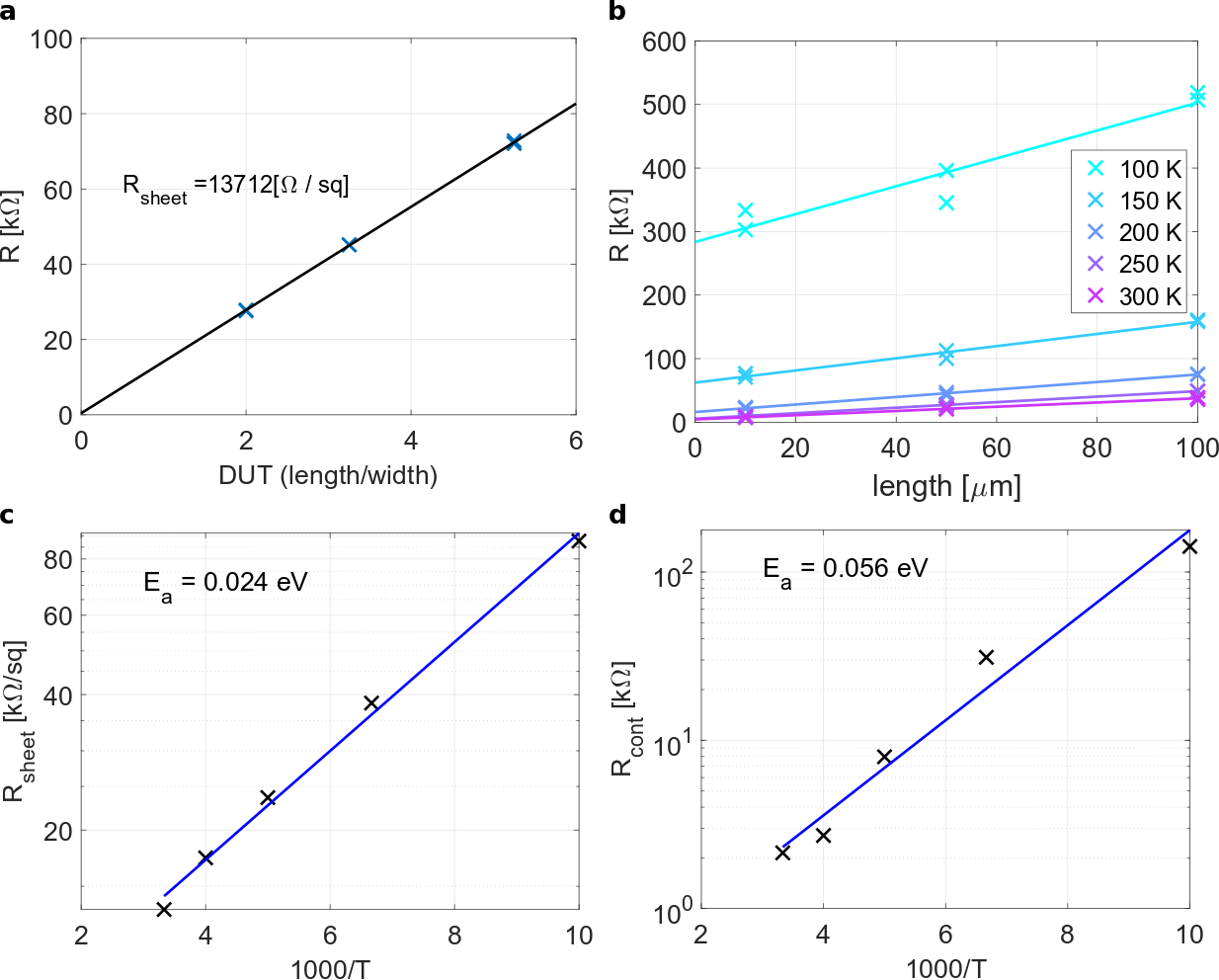


Figure S9 **Metal nitride sheet resistance** (a) 4 Probe resistance measured on bar structures at room temperature. (b) Temperature dependent resistance measured on two probe structures of different length. The data is fitted linearly to obtain the sheet resistance and contact resistance as a function of temperature (c&d). Both temperatue dependencies can be described by an Arrhenius behavior.

*Characterization of contact resistances to the metal electrodes*

*Metal electrode to PCM*

In our device model, we make some simplifications to the device geometry. In the real device, the phase change and projection material are patterned to a dog bone shaped geometry, whereas for the model we assume only a confined line in direct contact with the metal electrodes (Figure S10). Since the extended area of the dog bone does not participate in the switching process, its resistance is added to the resistor element that describes the contact to the metal electrode. The contact resistance to the electrode in the model is the sum of the material interface contact resistance between W and Sb, and the resistance of the extended area in the dog bone structure (e.g. R_W-Sb_ = R_cont(W-Sb)_ + R_patch(Sb)_).

R_W-Sb_ as defined here corresponds to the R_’cont’_ we defined to obtain the sheet resistance of crystalline antimony (Figure S7a). We measured a total contact resistance of 1622 Ω. To verify this value, we obtain R_cont(W-Sb)_ and R_patch(Sb)_ separately. Four probe contact resistance structures on the line-cell chip enable us to measure R_cont(W-Sb)_ directly for a contact area identical to the line-cell. We measure a resistance of 120±10 Ω. To obtain the R_patch(Sb)_ resistance, we perform a COMSOL-FEM simulation of an unprojected device with the geometry depicted in Figure S10. In the COMSOL simulation, R_cont(W-Sb)_ = 0 Ω. We calculate R_patch(Sb)_ = (R_sim_ – R_s,cryst_ * length/width)/2 = 1410 Ω, where R_sim_ is the total device resistance obtained in the COMSOL simulation, R­­_s,cryst_ the sheet resistance of crystalline Sb and length and width refer to the geometry of the line. The sum of the separately obtained R_cont(W-Sb)_ and R_patch(Sb)_ matches well with the directly measured value for R_W-Sb_. In our device model we use a contact resistance of 1622 Ω.


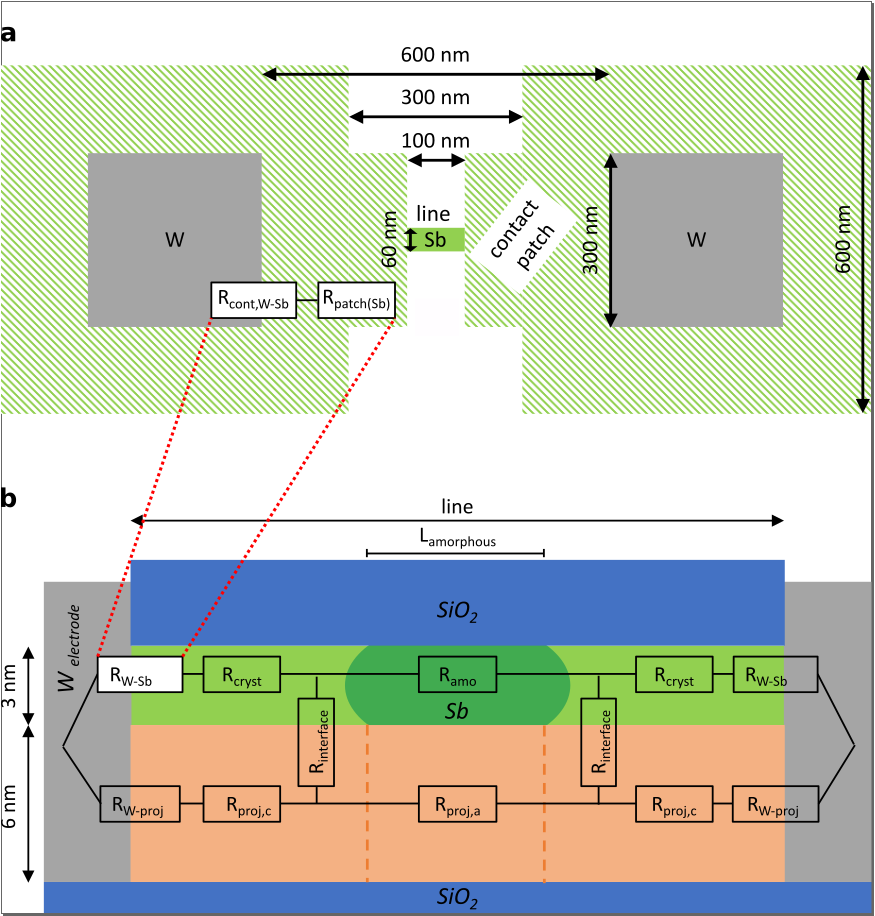


Figure S10 **Device geometry** (a) Top view sketch of the line-cell geometry. Antimony is marked in green, tungsten electrodes in grey. Only the central confined region of the device is the active volume in which material is melt-quenched and recrystallized. The resistance of the green striped area labled contact patch is in the device model (b) part of the resistor R_W-Sb_. In the model the PCM is reduced to the active region (line) of the device.

*Metal electrode to metal-nitride*

To define the resistor element R_W-proj_ in our equivalent circuit, we measure R_cont(W-proj)_ and R_patch(proj)_ separately. The contact resistance of W to metal nitride, R_cont(W-proj)_, was measured on micrometer scale reference structures in 4P configuration. The structures have a square contact area of varying size (75x75 um^2^; 50x50 um^2^ and 25x25 um^2^). The scaling of contact resistance with contact area can be described by the following formula

$R_{c}=\frac{\rho_{c}}{L_{t}*w}coth\left( L/L_{t} \right)$.

Here $\rho_{c}$ is the specific contact resistivity, L_t_ the transfer length, w the width of the contact area (perpendicular to the direction of current flow) and L the length of the contact area (in direction of current flow)[2]. For L > 1.5*L_t_, the coth converges to unity and R_c_ should scale with 1/w. If L < 0.5*L_t_, the coth(L/L_t_) can be approximated as L_t_/L. Accordingly, R_c_ would be proportional to the contact area (L*w). For our reference structures, R_c_ scales linearly with 1/w (Figure S11a). The slope tallies with $\rho_{c}$/L_t_. Since the transfer length is still an unknown variable, we can only estimate the contact resistance in our line-cells dependent on the transfer length (Figure S11b). The contact area is 300 x 300 nm^2^; i.e. L & w are 300 nm. We assume a transfer length in the range of 100 nm to 1 um, which corresponds to a contact resistance R_cont(W-proj)_ in the range of 18 kΩ (L_t_*1.5 < 300 nm) to 60 kΩ (L_t_ = 1 um) at 300 K. With the activation energy of 0.056 eV (Figure S9d) we extrapolate to a resistance R_cont(W-proj)_ of 53 kΩ to 177 kΩ at 200 K.

Like for the phase change material, the input parameter to our model R_W-proj_ is the sum R­_cont(W-proj)_ + R_patch(proj)_. The value R_patch(proj)_ = 25 kΩ is derived from the COMSOL simulation, with the same procedure described above for the metal to PCM interface. We estimate the input parameter R_W­-proj_ for our model to be in the range 78 kΩ to 202 kΩ.


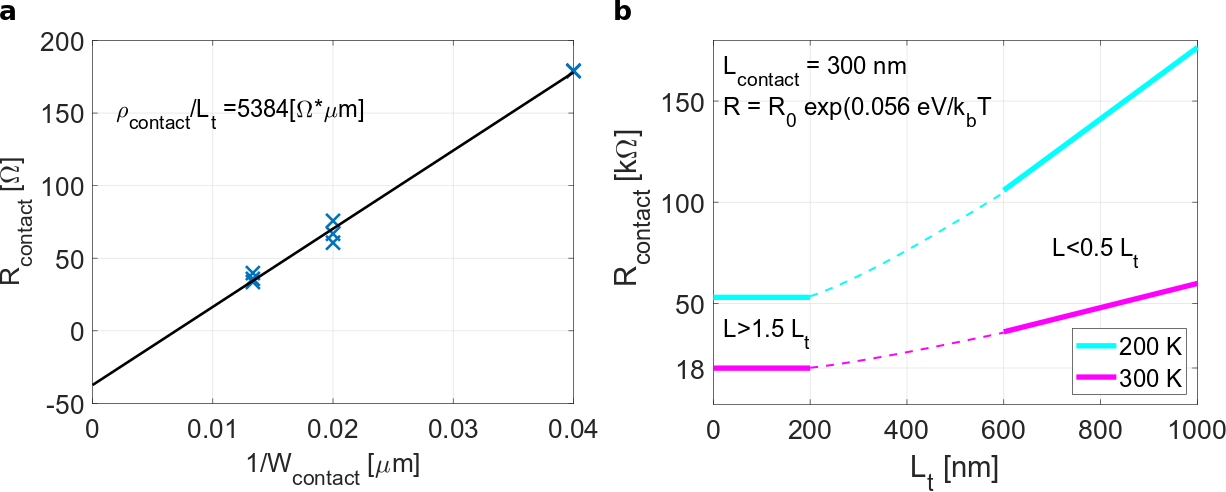


Figure S11 **Contact resistance W-metal nitride**(a) Four-Probe contact resistance measured on geometries with square contact area of varying size. The slope of the plot corresponds to the contact resistivity over transfer length [R_c_ ~ $\rho$_c_ /(w*L_t_) for L_contact_ > 1.5L_t_]. (b) Extrapolation of the contact resistance measured on reference structures to the 300x300 nm^2^ contact area in the line-cell. The contact resistance is a function of the transfer length.

1. **Drift Experiments**

*Drift measurement on micro to millisecond timescales*

On timescales from 50 µs to 700 ms after device RESET, we performed resistance measurements with an oscilloscope. We apply a burst of triangular pulses (13 us leading & trailing edge) with a 50 µs period and an amplitude of 0.2 V to capture an IV trace of the device. With this IV trace we obtain a measurement of the device resistance every 50 µs. The device current trace is amplified by a factor of 1000 with an DHPCA-100 Femto Current Amplifier. Before fitting the IV data, the current is passed through a lowpass filter with a cutoff frequency of 1 MHz to filter read noise (Figure S12 a).

The time dependent resistance data is processed with another smoothening filter (Savitzky-Golay finite impulse response smoothening filter) (Figure S12 b). This filter is applied for a clearer visualization and easier comparison of the different reset states created. Finally, the experimental data obtained in linear timescale is interpolated on a logarithmically spaced time grid with 200 points per order of magnitude. In this way, we assure an equal weight of the different timescales when the drift data is fitted to the model.
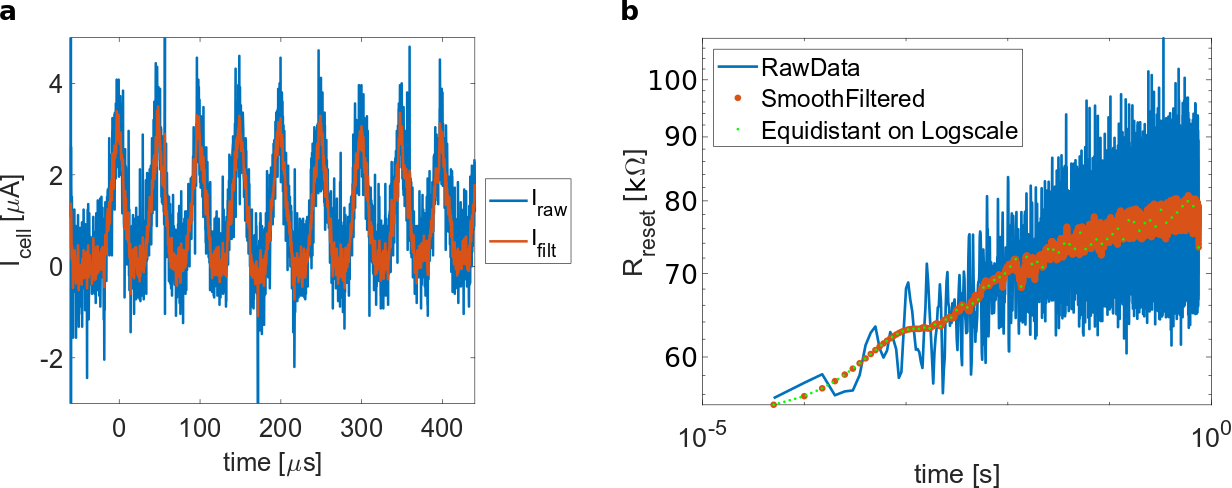


Figure S12 **Scope drift measurement – raw data** (a) Snippet of the current trace measured with the oscilloscope to obtain the time resolved device resistance. A burst of triangular voltage pulses (13 µs leading edge & trailing edge) is applied to the device with a 50 µs period. The current trace is processed with a 1 MHz lowpass filter. (b) Time resolved reset resistance. The raw data is smoothened and interpolated to a log spaced grid for further analysis and fitting to the device model.

*Temperature dependent resistance drift*

At an ambient temperature of 200 K, we measure a drift coefficient of 0.14+-0.01. This value is larger than the drift coefficient of 0.1±0.02 reported previously for an ambient temperature of 100 K [3]. Measurements on a single cell indeed confirm that the drift coefficient is lower at 100 K than 200 K (Figure S13).

_
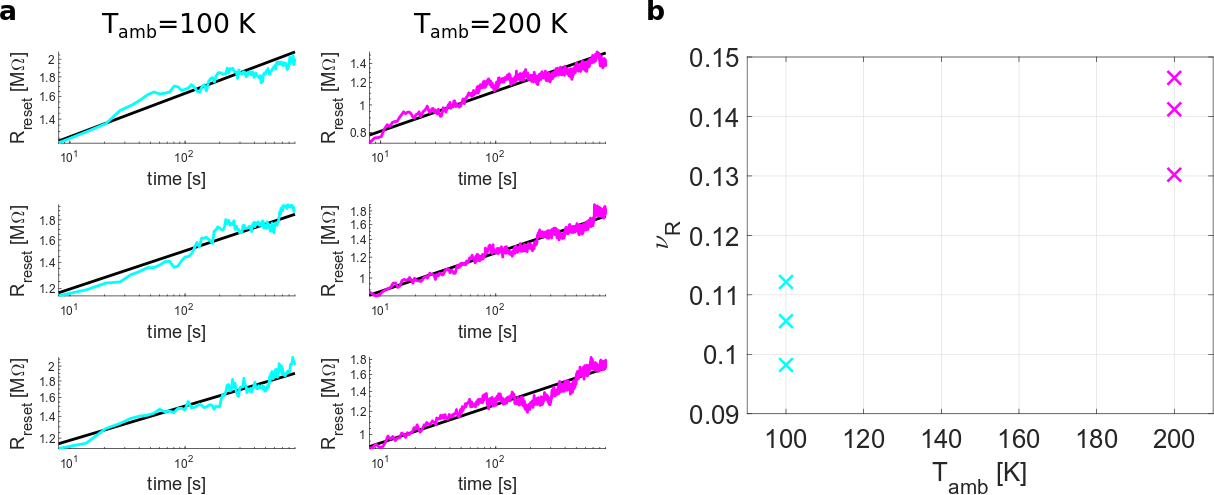
_

Figure S13 **Temperature dependent resistance drift** (a) Resistance drift measurement at 100 K and 200 K, all experiments are performed on the same device. Black lines show the fit to R = R_0_ (t/t_0_)^v^ (b) Drift coefficient as a function of ambient temperature.

*Additional drift measurements – unprojected*

We performed a total of 12 drift measurements on an unprojected memory cell (Figure S14a). The reset current to program the device was kept constant at 610 µA. Different reset states are created by varying the reset pulse trailing edge. The drift coefficient is independent of reset resistance and pulse trailing edge (Figure S14b).

_
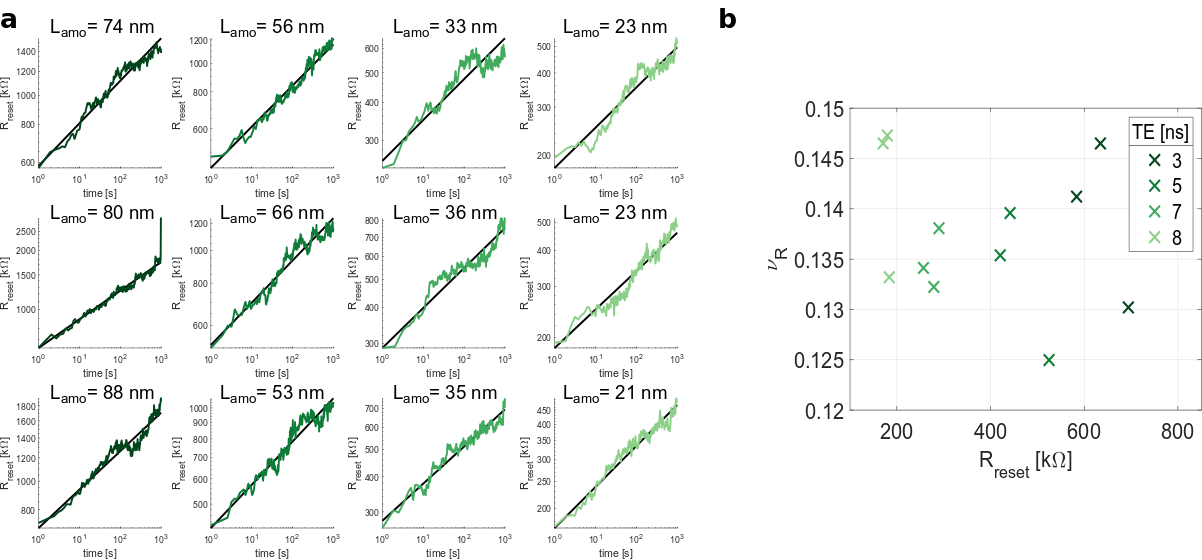
_

Figure S14 **Drift: Unprojected line-cell** (a) Temporal evolution of the reset resistance. The duration of the reset pulse trailing edge is color coded. Black lines show the fit to R = R_0_ (t/t_0_)^v^ (b) Drift coefficient.

*Additional drift measurements – projected*

We performed a total of 16 drift measurements on a projected memory cell (Figure S15). Different reset states are created by varying the reset pulse trailing edge. All experimental data sets are captured well by the model. The reset states created with the same pulse trailing edge are easily reproducible.

_
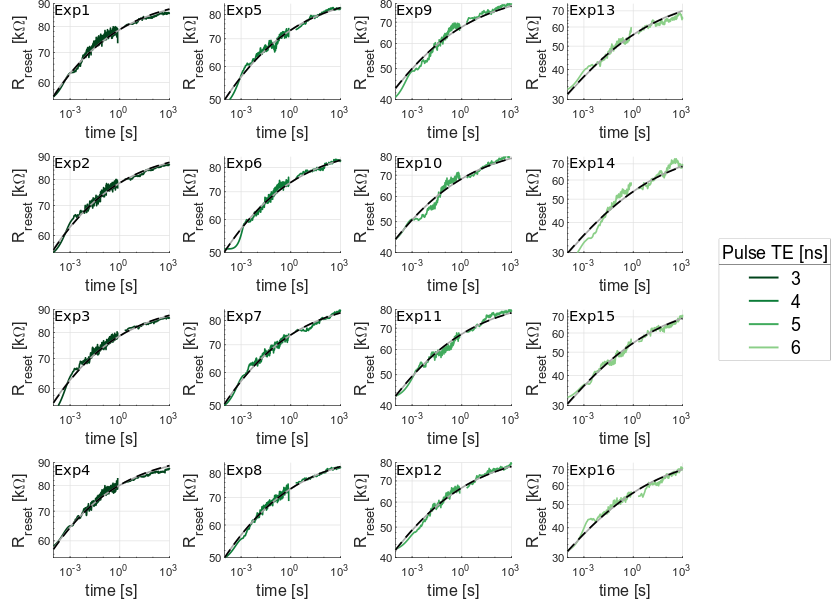
_

Figure S15: **Drift: Projected line-cell** Temporal evolution of the reset resistance. All measurements can be fitted well with our device model. Grey lines show the model fit with a contact resistance R_W-proj_ of 78 kΩ and dashed black lines with a contact resistance R_W-proj_ of 202 kΩ. These two values correspond to the estimated lower and upper bound of R_W-proj_.

Table 3: **Fitting parameter L_amo_**. Amorphous length for each reset state (Figure S11). The error margin is the deviation of fitting parameters if the system is solved for the upper and lower error margin of R_s,amo_ – Sb.

|  | L_amo_ | | | | | | | |
| --- | --- | --- | --- | --- | --- | --- | --- | --- |
| R_W-Proj_ [kΩ] | Exp1 | Exp2 | Exp3 | Exp4 | Exp5 | Exp6 | Exp7 | Exp8 |
| 78 | 51±6 nm | 52±7 nm | 51±6 nm | 54±6 nm | 40±5 nm | 40±5 nm | 41±5 nm | 40±5 nm |
| 202 | 49±5 nm | 49±6 nm | 49±6 nm | 52±6 nm | 39±5 nm | 39±5 nm | 39±4 nm | 39±4 nm |
| R_W-Proj_ [kΩ] | Exp9 | Exp10 | Exp11 | Exp12 | Exp13 | Exp14 | Exp15 | Exp16 |
| 78 | 30±4 nm | 31±4 nm | 29±4 nm | 28±4 nm | 16±3 nm | 15±2 nm | 15±2 nm | 17±2 nm |
| 202 | 29±4 nm | 30±4 nm | 28±3 nm | 28±4 nm | 16±2 nm | 15±2 nm | 15±2 nm | 16±2 nm |

1. **Threshold switching dynamics**

In this Supplementary Note we provide a definition of the threshold switching criterion and compare the switching dynamics in the projected and unprojected device. Both devices show abrupt threshold switching (Figure S16 a&b). The threshold switching IV characteristic, however, does not show a clear snap back upon threshold switching. For this reason, we use a threshold current level of I_th_ = V_cell_/R_reset_+12.5 µA to define the threshold voltage, instead of a snap back point. It defines the voltage level at which the device current significantly exceeds the ohmic current of the low-field resistance of the reset state. This metric enables us to define when both samples are switching and to derive the switching characteristic of the projected cells from the unprojected cells’ switching characteristics.

Assuming that the switching dynamics of the phase change material are not fundamentally changed by the projection layer, it should be possible to describe the switching of a projected device R_proj_(t) to first order by a switching PCM element R_PCM_(t) electrically in parallel to a constant projection element R_c,proj_ (Equation (8)). Note that this is a crude simplification of the full device model presented in Figure 1. To validate if this simplified picture is capable of describing the switching dynamics of a projected device, we calculated the transient resistance of the unprojected cell R_PCM_(t) and subsequently identified the projection resistor in parallel to the switching element. We compare the switching characteristics of a projected and an unprojected device for reset states with comparable threshold voltage. Here, we use a threshold current level of I_th_ = V_cell_/R_reset_ + 12.5 µA to define the threshold voltage (Figure S16 a and b). This metric enables us to define for both devices, with orders of magnitude different reset resistance, when they are switching.

|  | $R_{proj}\left( t \right)=\left( \frac{1}{R_{PCM,proj}(t)}+\frac{1}{R_{c,proj}} \right)^{-1}$ | (8) |
| --- | --- | --- |

The transient resistance of the unprojected cell R_PCM,unproj_(t) is calculated from the voltage and current trace measured with the oscilloscope, when the device is switched. The transient resistance obtained in this way is not reliable in the pre-switching region and at the end of the SET-pulse, because the measured device current fluctuates around zero in this range. In this range, we fix the resistance to the values measured with the source meter before and after switching the device respectively (Figure S16 c). From R_PCM,unproj_(t) we obtain the transient resistance of the phase change material in the projected device R_PCM,proj_(t) (Figure S16 d). To account for different device widths measured with TEM for the projected and unprojected devices, we scale the resistance by a physical width-based correction factor of 52 nm_unproj_/45 nm_proj_. Finally, we adjust the SET resistance level to the DC SET resistance measured for the projected device by adding an offset value. Small variations in the low resistive SET state result in a pronounced mismatch of measured and simulated IV characteristic for the projected device. These variations may arise due to inter device variability and cycle to cycle variability of the SET resistance. The offset correction accounts for this. The resulting transient resistance describes the resistance of the switching PCM element in the projected memory cell. With Equation 8 we compute the constant projection resistor in parallel to the switching element from the DC reset resistance of the projected and unprojected devices.

The phase change memory device R_proj_(t) is in series with an on-chip Tungsten resistor (3500 Ω) R_total_(t) = R_proj_(t) + 3500 Ω. In our model we calculate the threshold-switching current trace of the projected device (Figure S16 b) by dividing the voltage signal applied to the projected device by R_total_(t) (Figure S16 d). For three different reset states with threshold voltages around 1 V; 1.2 V and 1.35 V, the calculated switching IV of the projected cell matches the experimental results. This indicates that the threshold switching dynamics of the phase change material is not affected by the projection layer.

_
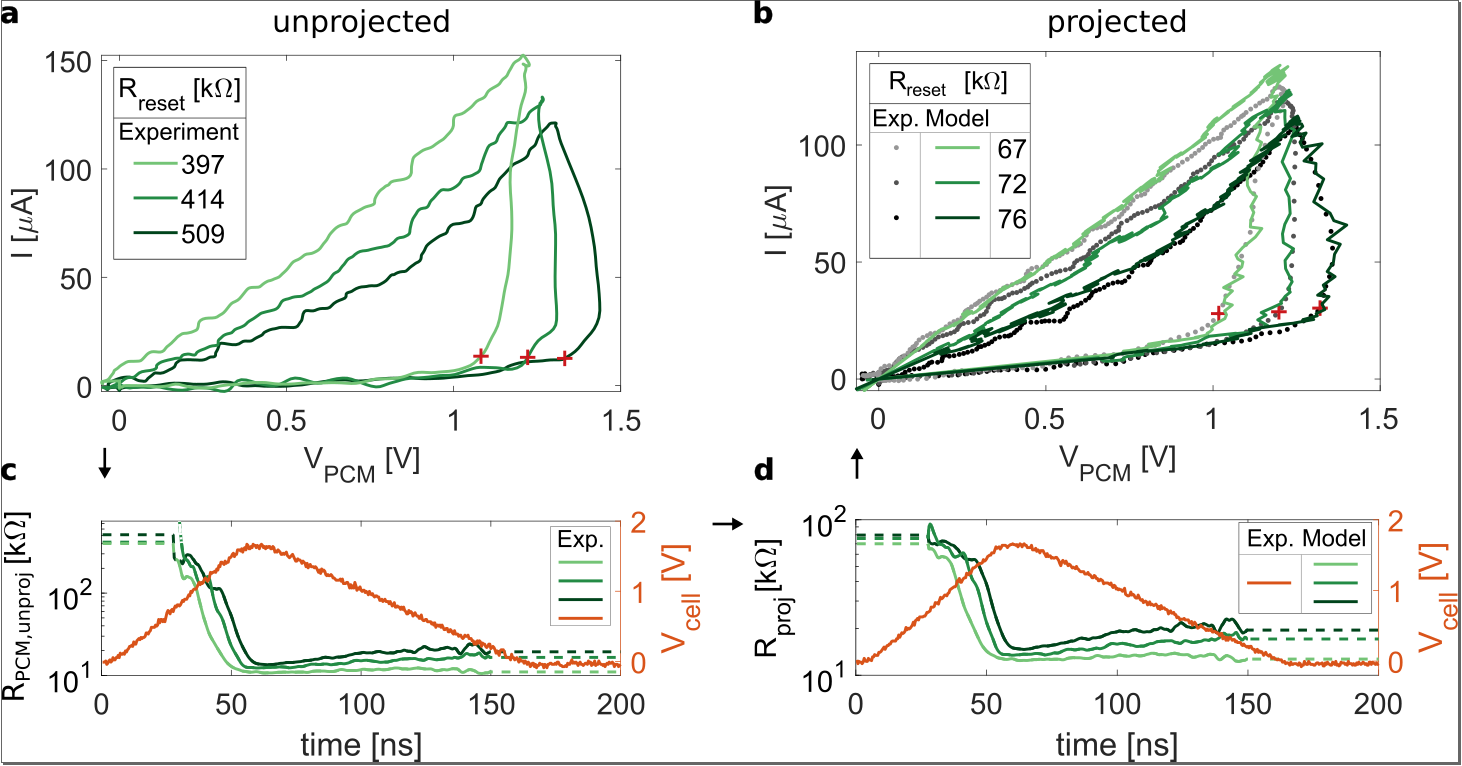
_

Figure S16: **Threshold switching IV characteristic of an unprojected (a) and projected device (b)**. We compare the threshold switching characteristic of both devices for RESET states with similar threshold voltage (marked by identical shades of green).The threshold voltage is marked by red crosses. It is defined at a threshold current level of I­_cell_ > V_cell_/R_reset_ +12.5 µA. The experimental switching IV-characterisitic of the projected device (dots in b) matches the model (lines). The transient resistance of the unprojected cell (c) is obtained experimentally. It is used to model the transient resistance of the projected cell (d).

1. **Threshold field**

The threshold voltage is a linear function of the amorphous length. V_th_ = E_th_*L_amo_ + V_offset_, where E_th_ denoted the threshold field and V_offset_ an offset voltage. For both, projected and unprojected device, we combine the data R_reset_(L_amo_) calculated with our device model with the experimental data R_reset_ (V_th_) to obtain the threshold field (Figure 4 in the manuscript). We find for both device a threshold field of 20±3 V/µm. The offset voltage increases from 0.27 V to 0.43 V. This difference in offset voltage may be an artefact of our threshold voltage definition.

Krebs et. al. reported a threshold field of 94±9 V/µm for as deposited amorphous Sb at room temperature [31]. We note that those as deposited samples have been heated to even higher temperatures during the device fabrication. Since the threshold field increases with structural relaxation, a higher value is to be expected for the as deposited amorphous samples than for the melt-quenched Sb measured here.

1. **Expansion on the constraints to define tolerable interface resistances**

*Maximum drift coefficient*

For finite interface resistances between phase change material and projection layer, the drift coefficient increases steeply for very small amorphous fractions. Our constraint requires all states with an amorphous fraction larger than 5% of the device length to have a drift coefficient smaller than 0.01. If the amorphous volume in the line-cell is very short compared to the width the probability of a crystalline percolation path becomes high. Such a state would have a low drift coefficient due to the percolation path. Amorphous phase configurations that do not block the complete line are not captured in our model.

*Restrict drift variability*

The separation between the most and least drifting device state must not change by more than 5% in the time window 1s to 10^4^ s. This criterion does not compare first coefficients, since these are changing state dependent with time. We consider the change of state separation a more defined and more tangible criterion. The criterion compares the most R∆_max_ and least R∆_min_ changing reset state. The separation between these two states is given by

|  | separation@1s = R∆_max_(@1s)‑R∆_min_(@1s) | (9) |
| --- | --- | --- |

and

|  | separation@1e4s = R∆_max_(@1e4s)‑R∆_min_(@1e4s). | (10) |
| --- | --- | --- |

The relative change of these two values must be smaller than 5%.

*Scaling of device resistance with the amorphous length*


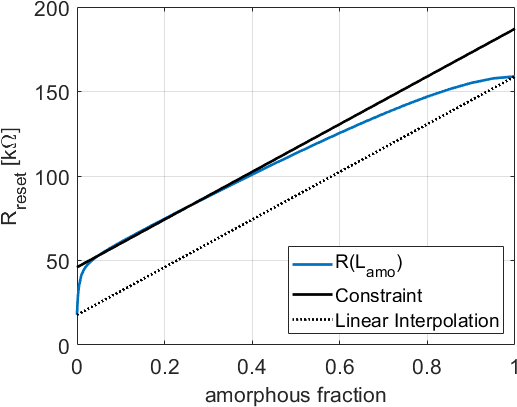
This criterion is supposed to limit the curvature of R(L_amo_). For this purpose, R(L_amo_) should not deviate by more than 20% from a linear interpolation between R_DUT,min_  and R_DUT.max_. The dynamic range is defined as R_DUT,max_ – R_DUT,min_. The curve R(L_amo_) is required to be smaller than the linear interpolation + 0.2 * the dynamic range. Figure S17 shows an exemplary data set R(L_amo_), limited by this constraint. R_reset_ states smaller 50 kΩ can most likely not be programmed reliably. The device resistance becomes very sensitive to the size of the amorphous length in this range. The rest of the programming range is less sensitive and can be addressed well.

Figure S17: **Example - constraint to limit the curvature of R(L­_amo_).** R(L_amo_) is calculated for R_s,cryst_ = 10 kΩ/sq, R_s,amo_ = 5000 kΩ/sq, R_s,proj_ = 80 kΩ/sq and R_int_ = 26 kΩ.

**References**

[1] M. Le Gallo, M. Kaes, A. Sebastian, and D. Krebs, “Subthreshold electrical transport in amorphous phase-change materials,” *New J. Phys.*, vol. 17, no. 9, p. 93035, 2015.

[2] D. K. Schroder, “MATERIAL AND DEVICE SEMICONDUCTOR MATERIAL AND DEVICE Third Edition,” *Phys. Today*, vol. 44, no. 4, p. 790, 2006.

[3] M. Salinga *et al.*, “Monatomic phase change memory,” *Nat. Mater.*, 2018.
